# Ufmylation‐Deficient DDRGK1 Ameliorates Obesity by Inhibiting FASN‐Mediated Adipocyte Lipogenesis

**DOI:** 10.1002/advs.202514702

**Published:** 2026-02-11

**Authors:** Yin Li, Tangjun Zhou, Xiao Yang, Kewei Rong, Xiankun Cao, Lei Shi, Xin Wang, Hongjin Wan, Lei Cui, Kexin Liu, Tong Xing, Hang Zhang, Chen Zhao, Tingxian Guo, Peixiang Ma, Jie Zhao, An Qin

**Affiliations:** ^1^ Shanghai Key Laboratory of Orthopedic Implants Department of Orthopedics Ninth People's Hospital Shanghai Jiao Tong University School of Medicine Shanghai China

**Keywords:** de novo lipogenesis, FASN, DDRGK1, UFMylation

## Abstract

Fatty acid synthase (FASN) is a central regulator of obesity through de novo lipogenesis (DNL), undergoes precise control via the ubiquitin‐proteasome system, yet its obesity‐related post‐translational modifications remain unclear. Our clinical investigation of human adipose tissue (n = 23) demonstrated elevated FASN protein in overweight individuals, mirroring high‐fat diet (HFD) mouse models. However, transcriptomic analysis of 770 GEO samples paradoxically revealed inverse correlation between *FASN* mRNA and BMI. Mechanistically, we identified DDRGK1 as a UFMylation effector that stabilizes FASN by competitively inhibiting ubiquitination. Genetic disruption of this pathway in *Ddrgk1^K268R^* mutant mice conferred metabolic protection, with 12% reduced body weight and 18% decreased fat mass under HFD conditions, alongside improved glucose homeostasis. Single‐nucleus RNA sequencing of inguinal white adipose tissue (iWAT) demonstrated reprogrammed metabolic flux in mutant mice, complemented by lipidomic profiling showing attenuated DNL. In vitro studies confirmed that DDRGK1 deficiency impairs adipocyte lipid droplet formation (reversible by palmitate acid supplementation) through FASN destabilization. Our work elucidates the adipocyte‐specific DDRGK1‐UFMylation‐FASN axis as a novel therapeutic target for obesity‐associated metabolic dysfunction.

## Graphical abstract

DDRGK1 regulates de novo lipogenesis via stabilization of fatty acid synthase (FASN). DDRGK1‐mediated UFMylation of FASN prevents its ubiquitin–proteasomal degradation. Reduced DDRGK1 expression or mutation at the key UFMylation site enhances FASN degradation and suppresses fatty acid synthesis (FAS), resulting in smaller adipocytes and improved insulin sensitivity. Therefore, the homeostasis of DDRGK1‐FASN interaction confers protection against diet‐induced obesity.

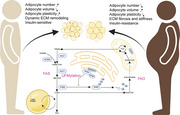

## 1 Introduction

During the progression of obesity, excessive expansion and hypertrophy of adipose tissue represent hallmark features and are primary contributors to the development of metabolic complications [1, 2, 3]. Adipose tissue is a specialized organ for storing excess energy as lipids and maintaining systemic metabolic homeostasis [4, 5, 6, 7]. Within adipocytes, DNL serves as a central pathway for synthesizing palmitate and its derivatives [8, 9, 10, 11]. Overactivation of this pathway is a significant contributor to obesity and nonalcoholic fatty liver disease [12]. At the core of DNL is FASN, a key enzyme that catalyzes the conversion of acetyl‐CoA and malonyl‐CoA into palmitate, highlighting its critical role in lipid metabolism and energy balance [13].

FASN plays a critical role in both metabolic diseases and cancers, where tumorigenesis is often accompanied by metabolic reprogramming, particularly an increase in DNL. Consequently, FASN is highly expressed in many tumors and represents a promising therapeutic target [14]. In colorectal cancer, fatty acid binding protein 5 is significantly downregulated and interacts with FASN, activating the ubiquitin‐proteasome pathway, which reduces FASN protein level and lipid accumulation [15]. In metabolic diseases like obesity, FASN also shows therapeutic potential. Hepatic *Fasn* knockout protects *ob/ob* mice against hepatic steatosis and improves glucose tolerance but exacerbates postprandial hyperglycemia and liver dysfunction, likely due to associated endoplasmic reticulum (ER) stress, inflammation and apoptosis [16]. The combination of FASN inhibitors and dietary polyunsaturated fatty acids has been shown to reduce triglyceride accumulation in the livers of HFD mice [17].

FASN undergoes a diverse array of post‐translational modifications, including methylation [18], palmitoylation [19], acetylation [20], lactylation [21] and ubiquitination [22]. High‐intensity interval training increases FASN lactylation, reducing its activity and DNL [21]. TRIM56, an E3 ubiquitin ligase, has also been implicated in regulating FASN. Hepatocyte‐specific deletion of *Trim56* exacerbates the progression of non‐alcoholic fatty liver disease by impairing FASN degradation through K48‐linked ubiquitination [23]. Previous studies have reported a negative correlation between *FASN* mRNA levels and obesity [24, 25, 26, 27, 28, 29, 30, 31, 32]. However, the precise mechanisms underlying FASN function in adipocytes, as well as its interplay with other post‐translational modifications such as UFMylation, remain largely unexplored. Comprehensive investigations are required to elucidate these mechanisms and to refine therapeutic strategies targeting FASN in metabolic disorders.

Apart from the aforementioned post‐translation modification, current understanding of UFMylation in metabolic diseases is limited, but emerging evidence highlights its importance. UFMylation is a type of ubiquitin‐like modification with a tertiary structure and enzymatic pathway (involving E1, E2, and E3 enzymes) similar to ubiquitination, covalently attaching UFM1 to lysine residues of substrate proteins through enzyme‐catalyzed reactions [33]. UBA5, UFC1, and UFL1 have been identified as the E1, E2, and E3 enzymes in the UFMylation pathway [34]. DDRGK1 (also known as UFBP1 or C20orf116), UFL1 and CDK5RAP3 form a tripartite complex that not only serves as a substrate for UFMylation, but also acts as a key regulator of UFMylation for other proteins [35, 36]. Studies have shown that the hepatic levels of UFL1 protein are reduced in *ob/ob* mice and HFD‐induced obese mice [37]. Mice with liver‐specific knockout of *Ufl1* or *Ddrgk1* develop steatohepatitis [38]. Conversely, upregulation of DDRGK1‐mediated UFMylation has been reported to alleviate non‐alcoholic fatty liver disease, improving hepatic steatosis and insulin resistance by mitigating ER stress [39]. These findings suggest that UFMylation plays a crucial role in maintaining metabolic homeostasis. However, the relationship between the UFMylation and obesity, lipid metabolism and DNL remains unclear, particularly given the lack of reported metabolic phenotypes associated with the knockout of its critical UFMylation sites. This highlights the need for further investigation to elucidate its function and metabolic implications.

In this study, we identified the interaction between FASN and DDRGK1, demonstrated that FASN is a substrate of UFMylation, and showed that UFMylation reduces its ubiquitination‐mediated degradation. Furthermore, we demonstrated that the *Ddrgk1^K268R^* mutation induces metabolic reprogramming in adipocytes and improves glucose tolerance and insulin sensitivity in HFD‐induced obese mice. Consistently, adipocyte‐specific *Ddrgk1* knockout mice exhibited reduced body weight and fat mass. These findings provide critical insights into adipocyte biology and suggest novel strategies for restoring metabolic homeostasis in obesity.

## 2 Results

### 2.1 UFMylation Component DDRGK1 Interacts With Lipogenesis Protein FASN

**FIGURE 1:**
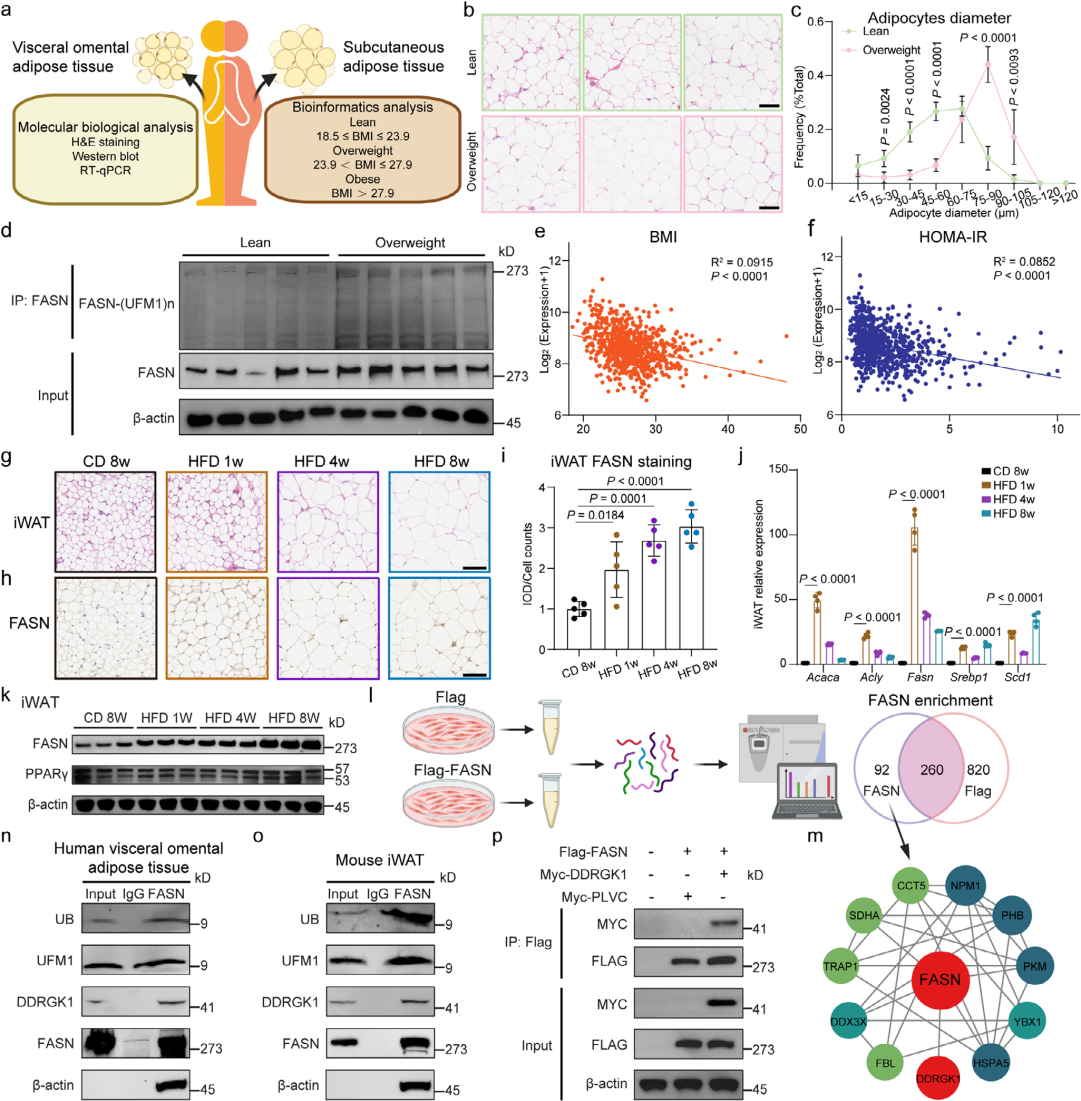
Identification of DDRGK1 as a FASN target in adipose tissue. (a) Molecular and bioinformatic characterization of FASN in human adipose tissue. *Left*: H&E staining, Western blot and RT‐qPCR analyses of FASN in omental adipose from 23 patients stratified by BMI (normal‐weight, n = 12; overweight, n = 11). *Right*: analysis of *FASN* mRNA expression (GSE70353 dataset; n = 770 subjects grouped by BMI). (b) Representative H&E‐stained omental adipose sections (Scale bars, 100 µm). (c) Quantitative measurement of adipocyte diameter (normal‐weight vs. overweight). (d) Western blot analysis of FASN protein and its ufmylation in omental adipose tissue from human subjects. (e,f) Correlation analyses between *FASN* expression and metabolic parameters: body mass index (BMI; e) and homeostatic model assessment of insulin resistance (HOMA‐IR; f). (g) Representative adipocyte morphology in iWAT from chow diet (CD)‐ and HFD‐fed mice (H&E staining; Scale bars, 100 µm). (h,i) Immunohistochemical detection of FASN protein in iWAT (Scale bars, 100 µm) and its quantitative analysis (i). (j) RT‐qPCR analysis of lipogenic gene expression in iWAT (n = 4 independent experiments, each with two technical replicates). (k) Western blot analysis of FASN and PPARγ in mouse iWAT. (l,m) Identification of FASN‐interacting proteins by immunoprecipitation followed by mass spectrometry (IP‐MS) in HEK293T cells. (n) Western blot of endogenous immunoprecipitation from human omental adipose tissue using FASN as the bait antibody. (o) Western blot of endogenous immunoprecipitation from mouse iWAT using FASN as the bait antibody. (p) Validation of the FASN‐DDRGK1 interaction by reciprocal Co‐IP. Panels (g), (l), and (m): One‐way ANOVA followed by Tukey's multiple comparison. Panels (c), (e), and (f): Two‐tailed unpaired Student's *t*‐tests. Panels (h) and (i): Pearson correlation with two‐tailed *P* values. Schematics in (a), (o) created with BioRender.com.

Our investigation of FASN regulation in obesity revealed a striking discordance between mRNA and protein expression patterns (Figure 1a). Analysis of omental adipose tissue samples from 23 individuals (12 lean and 11 overweight) revealed that overweight subjects displayed markedly enlarged adipocytes (Figure 1b,c) and increased levels of both FASN protein and its UFMylation modification (Figure 1d; Extended Data Figure S1a), despite a concomitant reduction in *FASN* mRNA expression (Extended Data Figure S1b). This inverse relationship was corroborated in a larger cohort analysis of 770 subcutaneous adipose samples (GSE70353 [40]). Among the samples, *FASN* mRNA level shows a significant downward trend (Extended Data figure S1c). The correlation between *FASN* mRNA expression and BMI, with HOMA‐IR showing a weak negative correlation (R = −0.303, *p* < 0.0001; R = −0.291, *p* < 0.0001) (Figure 1e,f).

In contrast to the discordant regulation of FASN observed in human adipose tissue, mice subjected to a HFD exhibited coordinated metabolic alterations characteristic of obesity progression. These changes were characterized by marked increases in body weight and adipose tissue mass (Extended Data Figures S2a–c), pronounced adipocyte hypertrophy (Figure 1g), and concurrent elevations in FASN protein abundance (Figure 1h,i,k) as well as in the expression of lipid metabolism‐related genes (Figure 1j; Extended Data Figure S2d).

To elucidate mechanisms underlying FASN protein stabilization, we performed mass spectrometry analysis following FASN overexpression and identified 92 interacting proteins (Figure 1l). Among these, ACLY, MAPK1, and CDK4 are known to be functionally related to FASN [41, 42, 43] (Figure 1m). FASN was newly identified as a DDRGK1‐interacting protein (Extended Data. Figure S3a) [44]. DDRGK1 is a key scaffold of the UFM1‑conjugation system that protects substrates from proteasomal degradation [44, 45]. Endogenous immunoprecipitation of human omental adipose tissue and mouse iWAT, using FASN as the bait antibody, demonstrated that FASN interacts with DDRGK1, ubiquitin (UB) and UFM1 (Figure 1n,o). Reciprocal co‐immunoprecipitation (Co‐IP) assays further confirmed a direct interaction between endogenous DDRGK1 and FASN in HEK293T cells (Figure 1p; Extended Data Figure S3b). Together, these data support DDRGK1 as an interacting protein of FASN.

### 2.2 UFMylation Stabilizes FASN and Inhibits Its Ubiquitination

The mammalian DDRGK1 sequence is highly conserved (Extended Data Figure S3b) [36]. DDRGK1 comprises 314 amino acids [46, 47]. Lysine 268 (267 in human) is critical for DDRGK1's UFMylation activity, forming a covalent link with UFM1 and UFL1 to create a Lys‐Gly crosslink [48]. We observed that DDRGK1 is abundantly expressed in adipose tissue, suggesting a role in adipocyte function (Extended Data Figure S3d–f).

**FIGURE 2:**
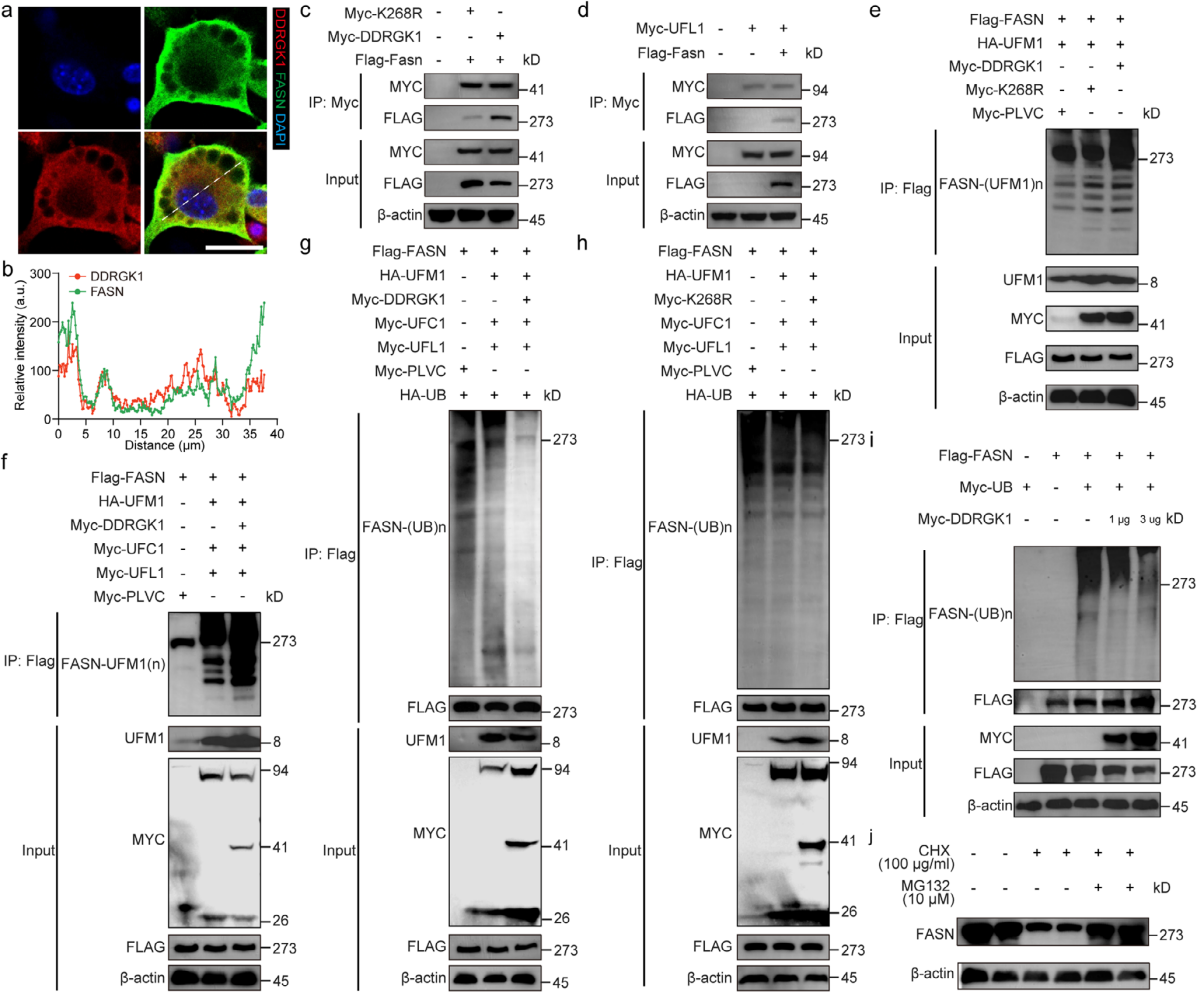
DDRGK1 interacts with FASN and inhibits its ubiquitination through UFMylation‐dependent mechanism. (a) Co‐localization of endogenous DDRGK1 (red) and FASN (green) in differentiated adipocytes. Nuclei were counterstained with DAPI (blue) (Scale bars, 20 µm). (b) Plots of pixel intensity along the white line from merged images. (c) Co‐immunoprecipitation (Co‐IP) demonstrating interaction between FASN and Myc‐tagged DDRGK1 or its mutant (K268R). Lysates from HEK293T cells transfected with indicated plasmids were immunoprecipitated with anti‐Myc beads. (d) Co‐IP analysis of Myc‐UFL1 and FASN interaction using anti‐Flag beads in HEK293T cells expressing Flag‐FASN. (e) UFMylation of Flag‐FASN in HEK293T cells co‐transfected with HA‐UFM1, Myc‐DDRGK1/K268R, and control vector (Flag‐PLVC). Immunoprecipitation was performed with anti‐Flag beads. (f) Complete UFM1 system (DDRGK1/UFL1/UFC1) maximizes FASN modification. HEK293T cells were transfected as in (e) with additional Myc‐UFL1 and Myc‐UFC1 plasmids. (g) Ubiquitination assays showing Flag‐FASN modification under UFMylation system activation (UFM1/DDRGK1/UFL1/UFC1) with Myc‐ubiquitin co‐expression. MG132 (10 µM) was added 8 h before lysis. (h) Control experiment demonstrating FASN ubiquitination is unaffected by K268R mutant under identical conditions as (g). (i) DDRGK1 alone reduces basal FASN ubiquitination. (j) Protein stability assays in adipocytes. Cycloheximide (CHX, 100 µg/mL) chase assay showing FASN and DDRGK1 protein stability in differentiated adipocytes treated with/without proteasome inhibitor MG132 (10 µM, 8 h) (n  =  3 biological replicates).

To elucidate the subcellular association between DDRGK1 and FASN, we performed immunofluorescence co‐localization analysis in differentiated adipocytes, which revealed overlapping DDRGK1 and FASN signals within the perinuclear and endoplasmic reticulum (ER) regions (Figure 2a,b). A similar co‐localization pattern was observed in HEK293T cells (Extended Data Figure 4a,b). Introducing the K268R mutation in DDRGK1 drastically reduced its ability to bind FASN (Figure 2c). We next tested UFL1 and UFM1, finding that both co‐immunoprecipitated with FASN (Figure 2d,e; Extended Data Figure S3a).

Ubiquitin‐like modifications, exemplified by UFMylation, serve as regulatory mechanisms that oppose ubiquitin‐mediated proteolysis [44, 49, 50]. DDRGK1 is a key UFMylation enzyme whose dysregulation associates with various pathologies. We used a multi‐plasmid co‐transfection system and found that FASN is regulated by ubiquitination (Figure 2f). Upon DDRGK1 overexpression, UFMylation of FASN was significantly enhanced, effectively conferring resistance to ubiquitination (Figure 2g). In contrast, overexpression of the Myc‐K268R plasmid failed to suppress ubiquitination (Figure 2h). Furthermore, a dose‐dependent regulation of FASN ubiquitination by DDRGK1 was observed when varying concentrations of the DDRGK1 plasmid were introduced (Figure 2i; Extended Data Figure S4c,d). Together, these results establish that DDRGK1‐mediated UFMylation is critical for maintaining FASN stability by inhibiting its ubiquitination and subsequent proteasomal degradation.

To assess the impact of DDRGK1 on FASN stability, we treated differentiated adipocytes with cycloheximide (CHX) and MG132. We observed that FASN was regulated by ubiquitination (Figure 2j). In order to further observe the changes of FASN in adipocytes with full knockout of *Ddrgk1* and the phenotype of mice, we generated inducible global knockout *Ddrgk1* mice (*Ddrgk1^CAGG‐ERT^*) and isolated SVF cells from the white adipose tissue. Tamoxifen was then applied in vitro to induce *Ddrgk1* knockout in these cells (Extended Data Figure S4e,f). Further studies have found that the degradation of FASN is mainly regulated by proteasome ubiquitination rather than lysosomal degradation (Extended Data Figure S4g–j).

### 2.3 *Ddrgk1* ^*K268R*^ Improves Metabolic Outcomes in HFD Mice

We further use *Ddrgk1^CAGG‐ERT^* mice to assess DDRGK1 role in metabolic homeostasis under HFD conditions. Global DDRGK1 knockout resulted in poor viability both before and after weaning (Extended Data Figure S5a,b), although HFD surprisingly extended survival (Extended Data Figure S5e,f). When *Ddrgk1* deletion was induced by tamoxifen, on either CD or HFD fed mice exhibited significant reductions in body weight and fat mass, demonstrating DDRGK1's critical role in systemic metabolism (Extended Data Figure S5c,d, g,h). Because knockout mice could not endure prolonged HFD feeding, we focused subsequent analyses on the *Ddrgk1^K268R^* mice.

**FIGURE 3:**
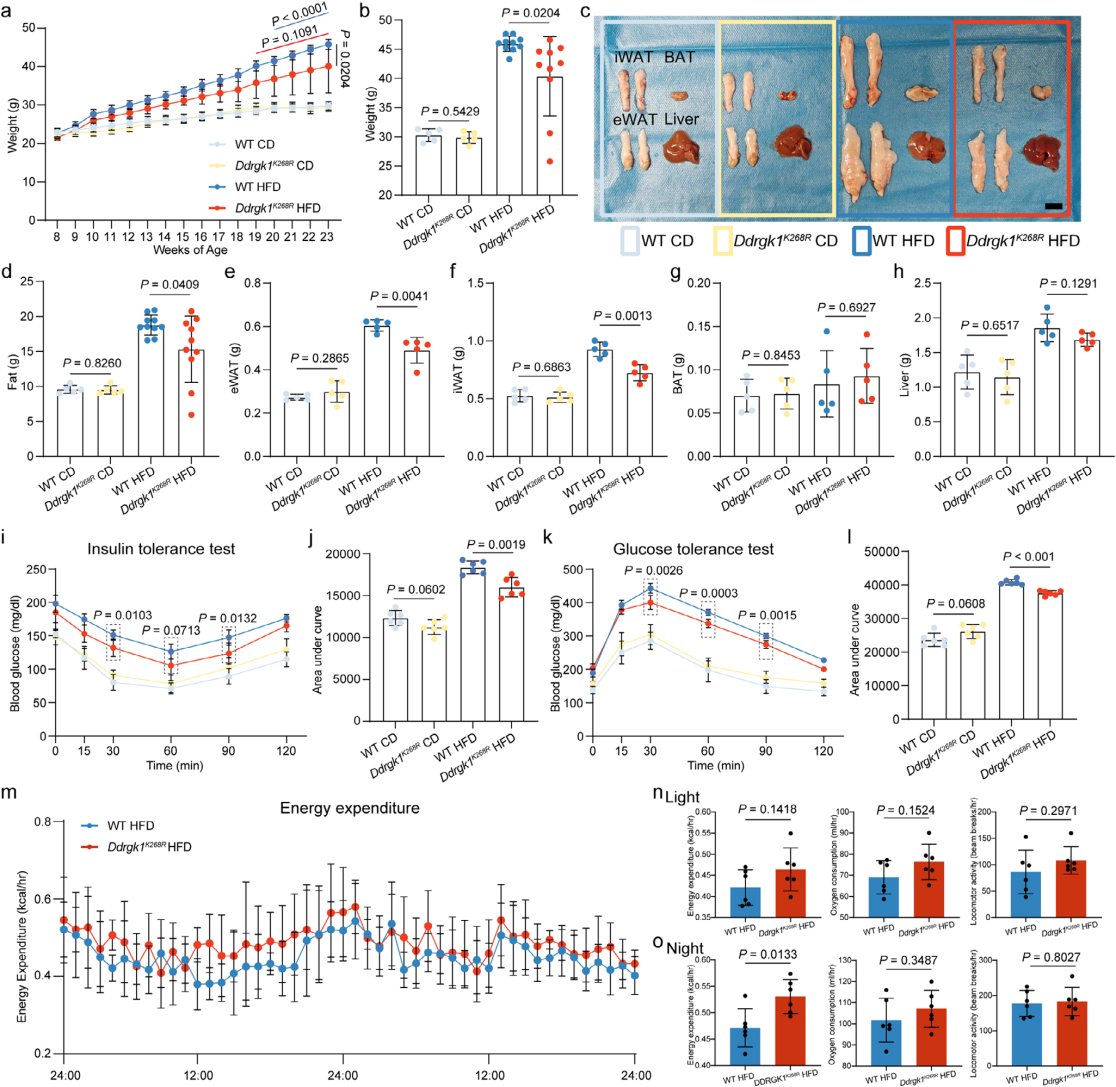
*Ddrgk1^K268R^* mutation protects against HFD‐induced obesity in mice. (a–c) Metabolic characterization of 23‐week‐old male mice fed CD or HFD. (a) Longitudinal body weight measurements from weeks 8–23. (b) Terminal body weight at week 23. (c) Representative images of gross morphology of epididymal white adipose tissue (eWAT), iWAT, brown adipose tissue (BAT) and liver (Scale bars, 1 cm). Group sizes: n = 5 (WT CD, *Ddrgk1^K268R^* CD), n = 10 (WT HFD, *Ddrgk1^K268R^* HFD). (d–h) Tissue weight analysis. (d) Total fat mass. (e) eWAT weight. (f) iWAT weight. (g) BAT weight. (h) Liver weight. (i–l) Metabolic tolerance tests. (i) GTT with 2 g/kg glucose injection. (k) ITT with 0.75 U/kg insulin. Area under the curve (AUC) values are shown (j, l). n = 6 per group. (m–o) Comprehensive metabolic cage analysis during 2‐day HFD challenge. (m) Energy expenditure (EE) during light and dark phases. (n) Energy expenditure, oxygen consumption and locomotor activity during the light phase. n = 6 per group. (o) Energy expenditure, oxygen consumption and locomotor activity during the night phase. n = 6 per group. All data represent mean ± s.d. Statistical significance was determined by two‐tailed unpaired Student's t‐tests.

**FIGURE 4:**
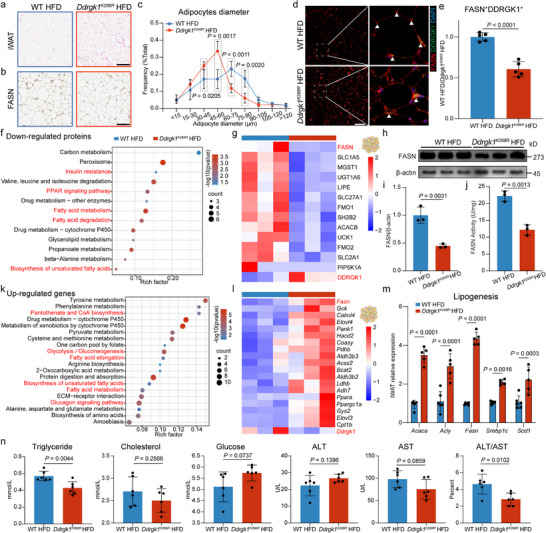
*Ddrgk1^K268R^* enhances systemic metabolism in HFD‐fed mice through lipogenic pathway modulation. (a–d) Adipose tissue characterization. (a) Representative H&E staining of iWAT showing adipocyte morphology (Scale bars, 100 µm). (b) FASN immunohistochemistry (brown staining) in iWAT sections. (c) Quantified adipocyte size distribution from H&E images. n = 5 per group. (d) Co‐localization of DDRGK1 (green) and FASN (red) in iWAT by immunofluorescence (Scale bars, 100 µm). Nuclei counterstained with DAPI. (e) Quantitative analysis of DDRGK1^+^FASN^+^ co‐localized area (% total area) from panel (d). (f) KEGG pathway enrichment of downregulated proteins by proteomics. (g) Heatmap of significantly downregulated lipogenic proteins and DDRGK1. (h,i) Validation of FASN protein levels. (h) Western blot of FASN in iWAT. (i) Quantified FASN protein levels normalized to β‐actin. (j) FASN activity of iWAT. n = 3 per group. (k) KEGG pathway enrichment of upregulated genes by transcriptomics analysis. (l) Heatmap of significantly upregulated lipogenic genes and *Ddrgk1*. (m) mRNA expression of lipogenic genes (*Acaca*, *Acly*, *Fasn*, *Srebp1c* and *Scd1*) measured by RT‐qPCR, normalized to 36B4 and presented as fold‐change relative to WT HFD (mean ± s.d., n = 6 mice with triplicate technical replicates). (n) Serum metabolic parameters: triglyceride, cholesterol, glucose, aspartate aminotransferase (AST), alanine aminotransferase (ALT) and ALT/AST ratio. n = 6 per group. All data were shown as the mean ± s.d. The statistical significance was calculated using two‐tailed unpaired Student's *t*‐tests.

After 15 weeks of HFD, *Ddrgk1^K268R^* mice displayed lower total body weight, fat mass, individual fat‐pad weights and liver mass compared with WT controls (Figure 3a–h). Glucose tolerance and insulin sensitivity were improved in mutants, as shown by intraperitoneal glucose tolerance test (GTT) and insulin tolerance test (ITT) (Figure 3i–l). Besides, metabolic cage studies revealed that *Ddrgk1^K268R^* mice exhibited higher energy expenditure at night (Figure 3m–o). To control for potential confounding effects of body composition, we conducted an ANCOVA with fat mass as a covariate. This analysis demonstrated that energy expenditure, oxygen consumption and carbon dioxide production remained significantly elevated in the experimental group, indicating that these metabolic differences are independent of adiposity. Histological analysis of adipose tissue in *Ddrgk1^K268R^* mice revealed smaller adipocytes (Figure 4a–c), reduced fat volume and decreased FASN^+^DDRGK1^+^ regions (Figure 4d,e), highlighting the mutation's impact on adipocyte morphology and metabolism. Smaller adipocytes generally indicate “healthy” obesity and are related to insulin sensitivity [3, 51].

To elucidate the mechanism of *Ddrgk1^K268R^* in white adipose tissue, we integrated proteomics and transcriptomics of iWAT and focusing on downregulated proteins and upregulated genes. For proteomics, KEGG enrichment analysis of significantly downregulated proteins in *Ddrgk1^K268R^* mice highlighted pathways related to lipid metabolism, including insulin resistance, PPAR signaling pathway and fatty acid metabolism (Figure 4f). The heatmap and western blot analysis indicated a decrease in FASN protein levels (Figure 4g–i). Other UFMylation components and related proteins, including UFM1, UFL1, UFC1, p53, CYB5R3, CDK5RAP3 and UFBP1, showed no significant changes. This may be because these proteins do not undergo the substantial alterations observed for FASN during adipocyte enlargement, making their stability less susceptible to perturbation. Consistently, enzymatic activity assays revealed a marked reduction in FASN activity in the iWAT of *Ddrgk1^K268R^* mice, paralleling the decrease observed at the protein level (Figure 4j). For transcriptomics, KEGG enrichment of significantly upregulated genes revealed enrichment in metabolic pathways such as pantothenate and CoA biosynthesis, glycolysis and gluconeogenesis and fatty acid elongation (Figure 4k). The heatmap and RT‐qPCR analysis indicated an increase in FASN mRNA level (Figure 4l,m). Additionally, biochemical analysis of serum revealed significant improvements in triglyceride and ALT/AST levels (Figure 4n). And lipid droplet staining analyses revealed decreased ectopic lipid droplet deposition in the liver of *Ddrgk1^K268R^* mice (Extended Data Figure S6d). In parallel, transcriptomic analyses of other energy‐metabolizing organs, including BAT and liver, revealed distinct patterns of differential gene enrichment. In BAT, the differentially expressed genes were primarily enriched in pathways such as “Complement and coagulation cascades,” “Retinol metabolism,” and “Steroid hormone biosynthesis” (Extended Data Figure S6e,f). In contrast, hepatic differential genes were predominantly associated with protein biosynthesis‐related pathways, including “Spliceosome,” “Protein processing in the endoplasmic reticulum,” and “Ribosome biogenesis in eukaryotes” (Extended Data Figure S6g,h). Heatmap analysis revealed tissue‐specific regulation of lipogenic genes across iWAT, BAT and liver in *Ddrgk1^K268R^* mice, with marked suppression in iWAT and divergent changes in BAT and liver (Extended Data Figure S6i).

Collectively, these findings suggest that *Ddrgk1^K268R^* induces adipose tissue metabolic reprogramming by modulating DNL, characterized by reduced fat mass, decreased ectopic lipid deposition, and improved systemic metabolic parameters, underscoring its therapeutic potential for obesity and metabolic disorders.

### 2.4 Single‐Nucleus Atlases of iWAT and Metabolic Analysis

**FIGURE 5:**
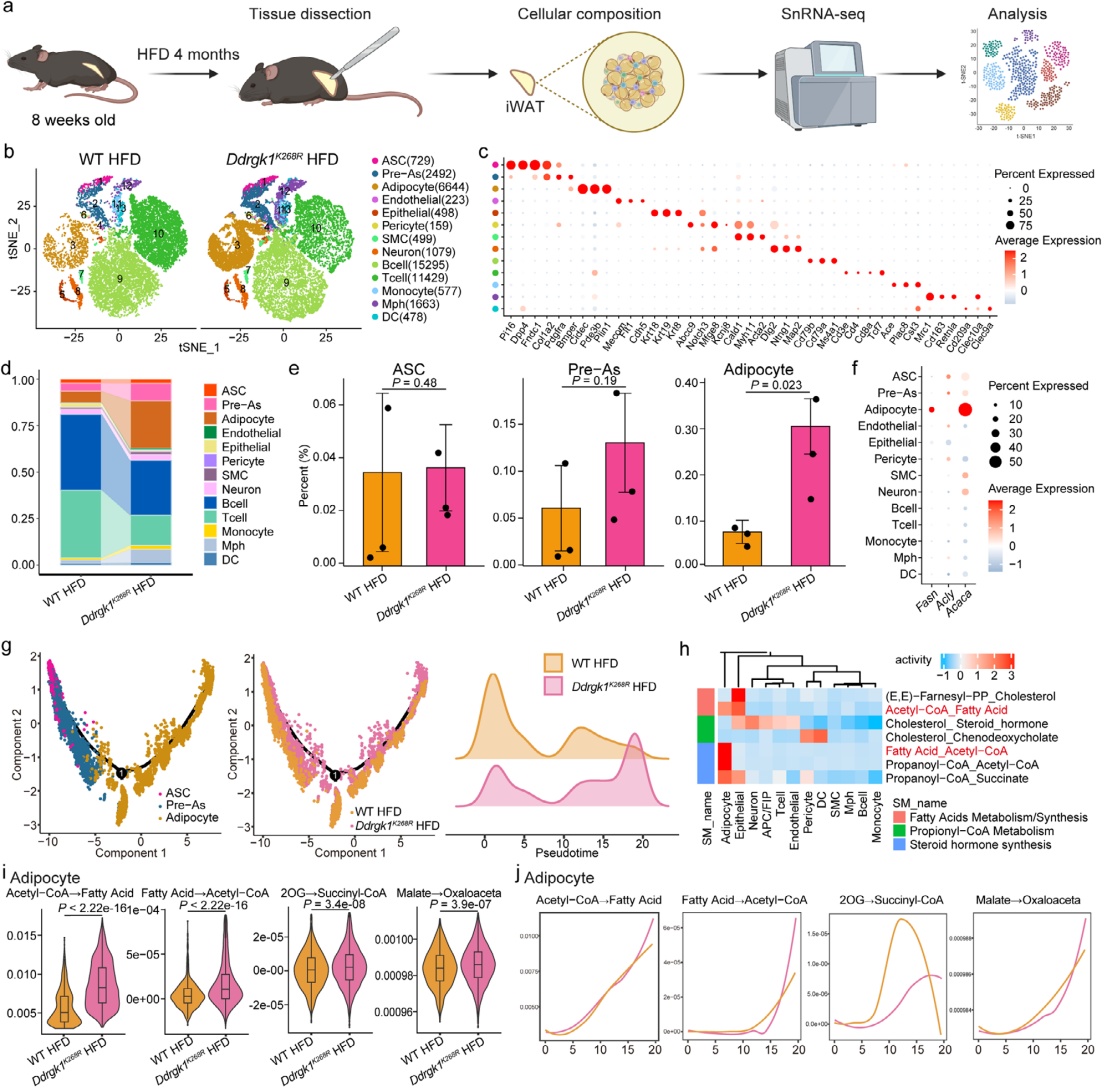
Single‐nucleus RNA sequencing reveals cell‐type‐specific metabolic reprogramming in iWAT of *Ddrgk1^K268R^* mice. (a) Experimental workflow. iWAT from 23‐week‐old HFD‐fed WT and *Ddrgk1^K268R^* mice (n = 3 biologically independent samples per group) was processed for snRNA‐seq (10× Genomics Chromium). Illustration created with BioRender.com. (b) Cell atlas characterization. T‐SNE projection of 12 annotated cell populations: adipocyte stem cells (ASCs), pre‐adipocytes (Pre‐As), mature adipocytes, endothelial cells, pericytes, smooth muscle cells (SMCs), neurons, B cells, T cells, monocytes, macrophages (MPHs) and dendritic cells (DCs). (c) Marker gene expression. Heatmap of top 3 differentially expressed genes (DEGs) per cluster (columns: genes; rows: cells). Color scale: log‐normalized counts. (d,e) Population distribution. (d) Stacked bar plot of cluster proportions per sample. (e) Relative abundance of major cell types between genotypes. (f) Lipogenic differential genes (*Fasn, Acly* and *Acaca*) expression of each cell types. (g) Lineage reconstruction. RNA velocity and Slingshot trajectory analysis of ASC differentiation (pseudotime 0–20 representing undifferentiated to mature states). (h,i) Metabolic flux prediction. (h) ScFEA‐predicted pathway activity differences. (i) Normalized activity scores for significantly altered metabolic pathways. (j) Temporal lipid metabolism changes. Heatmap of pathway activity differences during pseudotime progression in WT versus *Ddrgk1^K268R^* adipocytes.

To elucidate the effects of the *Ddrgk1* mutation on adipose tissue remodeling, we performed single‐nuclei RNA sequencing (snRNA‐seq) on iWAT from six mice: three *Ddrgk1^K268R^* and three WT controls subjected to 15 weeks of HFD (Figure 5a). TSNE and UMAP analyses revealed an increased abundance of adipocyte progenitors and mature adipocytes in the mutant adipose tissue, consistent with histological staining results (Figure 5b–e; Extended Data Figure S6a,b). Furthermore, snRNA‐seq showed elevated expression levels of three key enzymes involved in the adipose DNL pathway in the mutant group (Figure 5f; Extended Data Figure S6c). Differentially expressed gene analysis in mature adipocytes highlighted significant enrichment in pathways regulating protein metabolic processes (Extended Data Figure 6d). And the trajectory analysis indicated an increased transition from adipose stem cells to mature adipocytes in *Ddrgk1^K268R^* mice under HFD conditions (Figure 5g).

**FIGURE 6:**
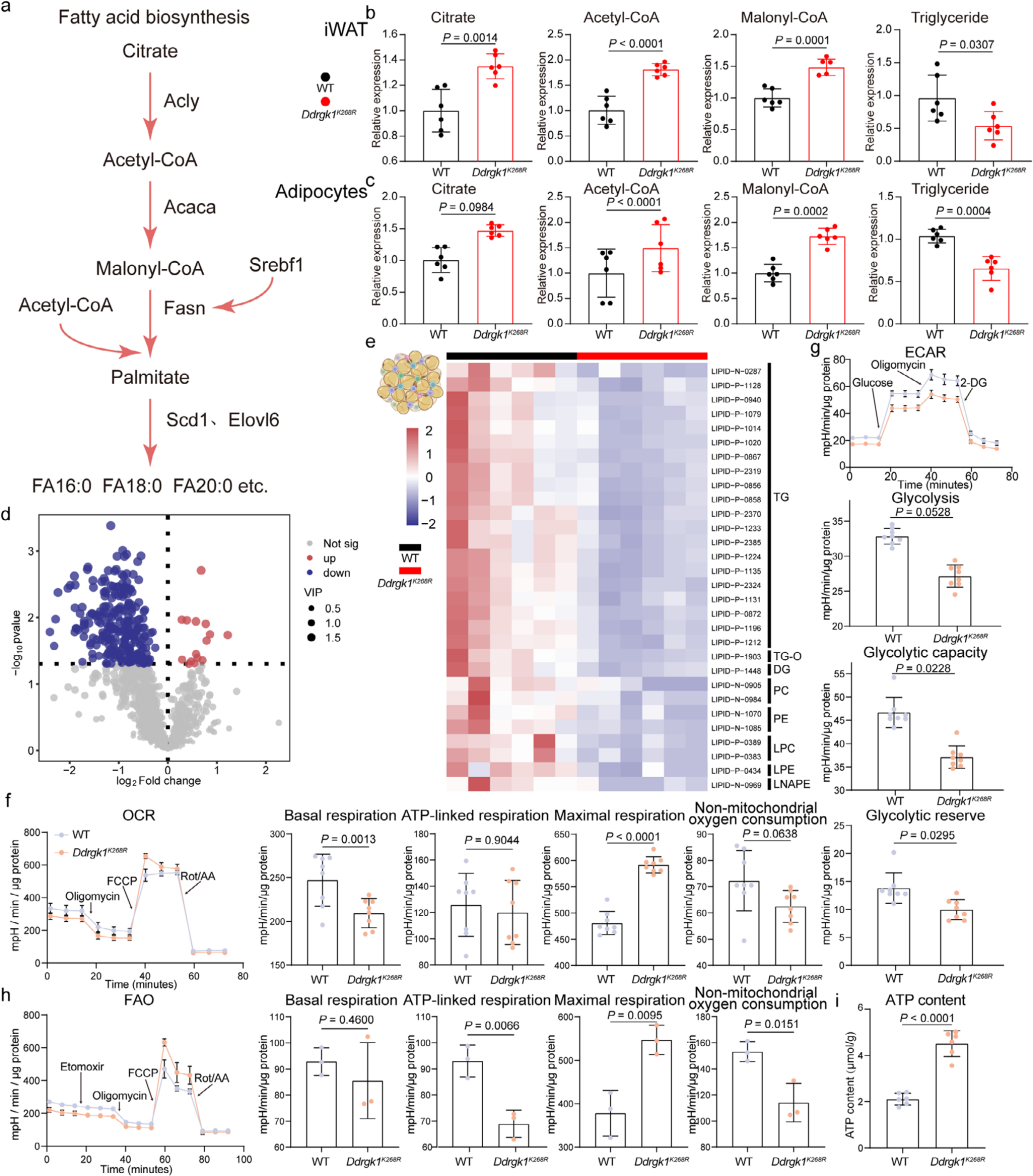
DDRGK1 regulates lipid deposition through DNL pathway modulation. (a) Schematic of DNL pathway highlighting key metabolites (citrate, acetyl‐CoA, malonyl‐CoA) and regulatory enzymes (Acly, Acaca, Fasn). Pathway diagram created with BioRender.com. (b,c) Metabolite quantification in iWAT tissue (b) and differentiated adipocytes (c) from WT versus *Ddrgk1^K268R^* mice: Citrate, acetyl‐CoA, malonyl‐CoA and triglyceride (normalized to tissue protein), n = 6 biologically independent samples. (d) Volcano plot showing significantly altered metabolites (red: upregulated; blue: downregulated; *p* ≤ 0.05, gray: nonsignificant). (e) Heatmap of significantly altered metabolites (rows) across samples (columns). Z‐score normalized. n = 6 per group. (f,g) Cellular bioenergetics. (f) Oxygen consumption rate (OCR, mpH/min/µg protein) measured by Seahorse XFe96 under basal/oligomycin/FCCP/rotenone conditions. n = 8 per group. (g) Extracellular acidification rate (ECAR, mpH/min/µg protein) reflecting glycolytic flux. n = 8 per group. (h) Oxygen consumption rate (FAO, mpH/min/µg protein) of differentiated adipocytes measured using the Seahorse XFe96 analyzer under sequential treatments with etomoxir/oligomycin/FCCP/rotenone. n = 3 per group. (i) Intracellular ATP content of differentiated adipocytes from WT and *Ddrgk1^K268R^* cells. n = 6 per group. All data were shown as the mean ± s.d. The statistical significance was calculated using two‐tailed unpaired Student's t‐tests.

To better understand the mutation's molecular impact, we computationally inferred cellular metabolic activities using a deep neural network model implemented in the scFEA Python package [52]. This analysis revealed mutation‐driven metabolic alterations, including shifts in lipid and glucose metabolism (Figure 5h). By comparing paired WT and *Ddrgk1^K268R^* samples, we examined whether our findings recapitulate known metabolic shifts during adipogenesis in *Ddrgk1^K268R^* mice (Figure 5i). During adipocyte differentiation, *Ddrgk1^K268R^* mice exhibited increased cellular metabolic flux, including enhanced lipid and glucose metabolism (Figure 5j). These findings suggest that the *Ddrgk1^K268R^* mice drives higher overall metabolic activity while reducing fat synthesis, thereby ameliorating HFD‐induced obesity.

### 2.5 Ddrgk1^K268R^ Decreases FASN Mediated Palmitate Synthesis

Given FASN role as the key enzyme in DNL, its reduced activity in *Ddrgk1^K268R^* mice led to a decrease in the downstream product palmitate and accumulation of upstream intermediates (Figure 6a). We quantified pyruvate, acetyl‐CoA, malonyl‐CoA and triglyceride in iWAT from WT and *Ddrgk1^K268R^* mice. All three upstream metabolites were elevated, while triglyceride level declined (Figure 6b). This pattern was recapitulated in cultured adipocytes (Figure 6c).

To profile lipid changes more broadly, we performed targeted lipidomic. *Ddrgk1^K268R^* mice showed significantly lower levels of palmitate derivatives (e.g., FA 16:0, FA 18:0) (Figure 6d,e), consistent with impaired FASN activity. Because DNL is related to the overall metabolism of cells, we next assessed cellular energy metabolism by seahorse assays in differentiated SVF cells. *Ddrgk1^K268R^* cells exhibited reduced extracellular acidification rates indicating suppressed glycolysis, and increased oxygen consumption rates, reflecting enhanced mitochondrial oxidative phosphorylation (Figure 6f,g). Fatty acid oxidation assays demonstrated that *Ddrgk1^K268R^* cells exhibit enhanced fatty acid oxidation capacity (Figure 6h), accompanied by an increase in intracellular ATP levels (Figure 6i). These results are fully consistent with the metabolic flux alterations inferred from our snRNA‐seq–based analysis.

Upon supplementation with exogenous palmitic acid (PA) (Extended Data Figure S8a), WT cells exhibited upregulation of both oxidative phosphorylation and glycolysis (Extended Data Figure S8c,d). Concurrently, we assessed metabolic changes in *Ddrgk1^CAGG‐ERT^* cells. These cells displayed elevated intracellular ATP levels (Extended Data Figure S8b). They displayed pronounced mitochondrial dysfunction, evidenced by decreases in mitochondrial oxidative phosphorylation, glycolysis and fatty acid oxidation capacity (Extended Data Figure S8e–g).

To further investigate whether *Ddrgk1^K268R^* affects the differentiation potential of SVF cells, RT‑qPCR analysis showed no significant changes in adipogenic gene expression in *Ddrgk1^K268R^* SVF cells compared with WT controls (Extended Data Figure S9a). In contrast, SVF cells from *Ddrgk1^CAGG‐ERT^* mice exhibited marked suppression of adipogenic gene expression (Extended Data Figure S9a). However, transcriptomic sequencing of differentiated WT and *Ddrgk1^K268R^* adipocytes identified 1886 differentially expressed genes (913 upregulated, 955 downregulated) (Extended Data Figure S9b). KEGG pathway analysis revealed enrichment in PPAR signaling, fatty acid metabolism and lipid degradation pathways (Extended Data Figure S9c). Key adipogenic genes, including *Fasn*, *Pparg* and *Fabp4* were upregulated in *Ddrgk1^K268R^* adipocytes (Extended Data Figure S9d). These findings were confirmed by RT‑qPCR, whereas differentiation of *Ddrgk1^CAGG‐ERT^* cells resulted in a pronounced downregulation of adipogenic genes (Extended Data Figure S9e).

Collectively, these results indicate that the *Ddrgk1^K268R^* impairs palmitate synthesis, reprograms adipocyte energy metabolism toward oxidative phosphorylation, and elicits compensatory transcriptional responses, underscoring its potential as a therapeutic target for obesity‐related metabolic dysfunction.

### 2.6 *Ddrgk1^K268R^* and *Ddrgk1^CAGG‐ERT^* Inhibit Lipid Droplet Accumulation

**FIGURE 7:**
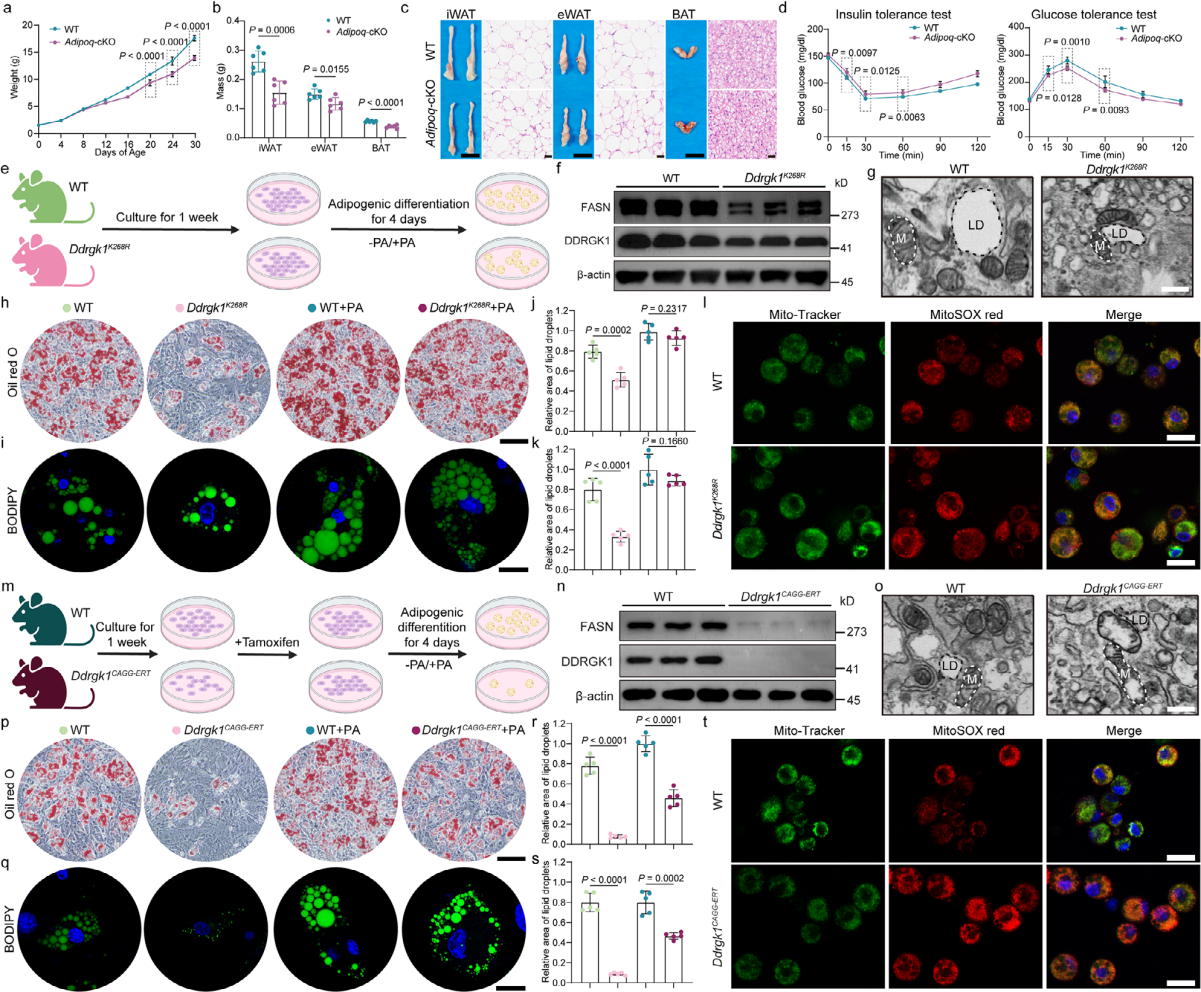
Reduced body weight and fat mass in adipocyte‐specific *Ddrgk1* knockout mice, accompanied by impaired lipid accumulation. (a) Targeting strategy for adipocyte‐specific deletion of DDRGK1. Exons are indicated by numbers, and triangles denote LoxP sites. WT: *Ddrgk1^flox/flox^*; *Adipoq*‐cKO: *Ddrgk1^flox/flox^*, *Adipoq*‐Cre. (b) Genomic DNA extracted from mouse tail biopsies was subjected to PCR using primers for the *Ddrgk1* ^flox^ allele and the *Adipoq*‐Cre transgene. Agarose gel electrophoresis revealed distinct band patterns corresponding to Flox^−/−^, Flox^+/+^, Cre^+^ and Cre^−^. (c) Western blot analysis of FASN and DDRGK1 protein levels in iWAT (β‐actin as loading control). (d) Relative *Ddrgk1* mRNA levels in iWAT detected by RT‐qPCR (WT and Adipoq‐cKO; n = 6 per group). (e) Longitudinal body weight measurements from days 0–30. (f) Tissue weight analysis: eWAT, iWAT and BAT. (g) Representative gross morphology (Scale bars, 1 cm) and H&E‐stained sections (Scale bars, 25 µm) of eWAT, iWAT and BAT. h, Metabolic tolerance tests. GTT and ITT. (i) Experimental workflow for SVF isolation and adipogenic differentiation from iWAT of WT and *Ddrgk1^K268R^* mice. Primary SVF cells were differentiated for 4 days, with palmitate (PA, 300 µM) or vehicle treatment starting on day 2 followed by lipid staining. (j) Western blot analysis of FASN protein levels in day‐4 differentiated adipocytes (β‐actin as loading control; quantification shown as mean ± SD, n = 3 independent differentiations). (k) Transmission electron microscopy of day‐2 differentiated adipocytes showing lipid droplets (LD, black arrowheads) and mitochondria (white arrowheads) (Scale bars, 1 µm). (l–o,t–w) Lipid droplet formation in differentiated SVF cells. (l,t) Oil Red O staining (Scale bars, 100 µm). (m,u) BODIPY staining (Scale bars, 25 µm). (n,o,v,w) Quantification of adipocyte differentiation by Oil Red O (% stained area) and BODIPY (% stained area) (n = 5 biological replicates). (p,x) Mito‐Tracker Green and MitoSOX Red double staining to assess mitochondrial function (Scale bars, 20 µm, n = 3 per group). Blue fluorescence: nuclei of live cells; green fluorescence: mitochondrial morphology; red fluorescence: oxidation product formed by MitoSOX Red. Panels (n,o,v,w): Two‐tailed unpaired Student's *t*‐tests. Schematics in (i,t) created with BioRender.com.

To more specifically delineate the role of DDRGK1 in adipose tissue, we generated adipocyte‐specific knockout mice (*Adipoq*‐cKO *Ddrgk1*) (Extended Data Figure S10a) and confirmed gene and protein deletion at both the transcript and protein levels (Extended Data Figure S10b‐d). *Adipoq*‐cKO mice were viable at birth and survived at least until the weaning stage (Figure 7a). *Adipoq*‐cKO mice exhibited smaller body size and reduced adipose mass, with pronounced reductions in eWAT, iWAT and BAT (Figure 7b). Histological analysis of iWAT and eWAT revealed smaller adipocytes (Figure 7c). Notably, *Adipoq*‐cKO mice displayed improved metabolic profiles, as evidenced by enhanced glucose tolerance and insulin sensitivity compared with wild‐type controls (Figure 7d). Collectively, these results indicate that DDRGK1 is critically required for adipose tissue development and maintenance and further corroborate our findings obtained from the whole‐body point‐mutant mouse model.

To assess DDRGK1 role in lipid droplet biogenesis, iWAT was isolated from *Ddrgk1^K268R^* mutants cultured in vitro and induced to differentiate into adipocytes (Figure 7e). 4 days post‐induction, *Ddrgk1^K268R^* showed markedly reduced FASN levels (Figure 7f). Consistent with this, oil red O and BODIPY staining revealed substantially fewer and smaller lipid droplets (Figure 7h‐k). To determine whether diminished palmitate production underlies impaired lipogenesis, we supplemented cultures with exogenous PA . Lipid droplet formation in *Ddrgk1^K268R^* cells was nearly fully rescued by PA (Figure 7h‐k). Transmission electron microscopy revealed no obvious morphological differences in mitochondria between *Ddrgk1^K268R^* and WT cells (Figure 7g), although *Ddrgk1^K268R^* cells exhibited slightly elevated mitochondrial ROS levels (Figure 7l).

Furthermore, we found that *Ddrgk1^CAGG‐ERT^* showed FASN decreased significantly 4 days after adipogensis (Figure 7m,n). Oil red O and BODIPY staining also revealed substantially fewer and smaller lipids (Figure 7p‐s). However, knockout cells showed only partial recovery after the addition of PA (Figure 7p‐s). Moreover, transmission electron microscopy revealed that complete loss of DDRGK1 resulted in pronounced mitochondrial structural abnormalities (Figure 7o), accompanied by a significant increase in mitochondrial ROS levels (Figure 7t).

These results indicate that total DDRGK1 deficiency broadly disrupts mitochondrial integrity and cellular metabolism, while the *Ddrgk1^K268R^* mutation specifically impairs DNL through reduced palmitate synthesis, a defect reversible by substrate replenishment.

## 3 Discussion

FASN is a pivotal enzyme in lipid synthesis and plays an essential role in lipid droplet accumulation [53], and its expression is therefore tightly regulated at both the transcriptional and translational levels. Multiple transcriptional regulators, including microRNAs [54], epigenetic modifiers [55] and lipid metabolism related transcription factors [56], have been shown to contribute to the control of *Fasn* expression under various metabolic conditions. In addition to transcriptional regulation, previous studies have highlighted the importance of post‐translational mechanisms. For example, work on FASN palmitoylation has demonstrated that this modification influences enzymatic activity and downstream signaling, including the interaction of FASN with mutant TP53, which enhances palmitoylation, reduces ubiquitination and promotes tumorigenesis [57]. However, the role of UFMylation in regulating FASN has remained unexplored. The UFMylation system is known to be crucial for ER functions, including ER autophagy and ribosome‐associated quality control [58], with genetic studies in mice revealing its importance in hematopoiesis, liver development, neurogenesis, and chondrogenesis [45, 59]. Despite these findings, its involvement in adipocyte metabolism has not been documented. Our study addresses this gap, demonstrating that DDRGK1 facilitates FASN UFMylation, stabilizing the enzyme and preventing its degradation via the ubiquitin‐proteasome pathway. Loss of *Ddrgk1* or *Ddrgk1^K268R^* reduced FASN UFMylation, increased its ubiquitination, and accelerated proteasomal turnover, thereby suppressing DNL. These findings expand the known scope of FASN post‐translational modifications and reveal that the balance between UFMylation and ubiquitination is a critical determinant of FASN stability in adipose tissue. Furthermore, this study is the first to establish a link between UFMylation and adipocyte metabolism, highlighting its pivotal role in the pathogenesis of obesity.

To date, researches on DDRGK1 in the context of metabolism have primarily focused on its role in ER stress signaling. DDRGK1, a critical ER‐associated protein [60], is a well‐established UFMylation substrate in bone and cartilage, where its dysfunction leads to skeletal dysplasia [61, 62]. Moreover, there is a well‐documented connection between lipid metabolism and the skeletal system [63], and recent studies suggest DDRGK1's involvement in non‐alcoholic fatty liver disease progression via ER stress modulation [37, 39]. However, the direct mechanisms by which DDRGK1 regulates metabolic enzymes have remained elusive. Our work identifies a novel mechanism wherein DDRGK1 directly binds FASN in adipocytes, regulating its post‐translational modifications and stability. Although *Ddrgk1^K268R^* mice have been studied in tumor models, their physiological and HFD phenotypes have not been explored [64]. Our data reveal that under normal physiological conditions, *Ddrgk1^K268R^* mice exhibit no significant differences in size or tissue structure compared to WT mice. However, when challenged with a HFD, *Ddrgk1^K268R^* mice alleviated obesity, with reduced body weight and fat mass compared to WT mice, while no significant differences were observed in adipose tissue inflammatory infiltration (Extended Data Figure S5a). This discovery highlights DDRGK1's physiologically relevant role in metabolic regulation, distinct from its ER stress‐related functions, and positions DDRGK1 as a key modulator of lipid metabolism.

Our study revealed a significant reduction in FASN protein levels within the iWAT of *Ddrgk1^K268R^* mice. Immunohistochemical analysis showed no genotype‐dependent differences in UCP1 protein (a hallmark of beige and brown fat [65]) expression in either iWAT or BAT (Extended Data Figure S6b,c), suggesting that *Ddrgk1* mutation does not overtly induce adipose being under HFD conditions. Previous studies have established that adipose tissue distribution and cellular composition are closely linked to metabolic health [66, 67], with alterations in adipocyte progenitor abundance, extracellular matrix organization, and stromal niche composition influencing adipocyte differentiation and metabolic function [68, 69, 70]. Consistent with this concept, our single‐nucleus transcriptomic analysis revealed a significant expansion of adipocyte progenitor populations in the iWAT of *Ddrgk1^K268R^* mice, indicative of altered adipose tissue remodeling. Moreover, scFEA‐based metabolic flux analysis uncovered a global upregulation of metabolic fluxes across multiple pathways, reflecting broad metabolic reprogramming rather than selective activation of a single metabolic route. Within this metabolic landscape, fluxes associated with fatty acid oxidation and the tricarboxylic acid cycle were elevated, consistent with enhanced oxidative metabolism. Importantly, these computational inferences were functionally supported by Seahorse‐based measurements.

Previous studies have shown that constitutive or inducible knockout of *Fasn* in adipocytes induces the emergence of beige adipocytes in iWAT, characterized by UCP1 expression and enhances systemic glucose tolerance and fatty acid oxidation [71, 72]. In *Ddrgk1^K268R^* mice and differentiated primary adipocytes, upstream metabolites such as acetyl‐CoA and malonyl‐CoA accumulated, while the synthesis of downstream products like palmitate and its derivatives was restricted. These findings align with the observed reduction in FASN protein levels [73, 74]. Acetyl‐CoA and malonyl‐CoA are not direct drivers of browning [75]. Acetyl‐CoA plays a dual role, with one fraction converted into fatty acids for energy storage and the other entering the TCA cycle to generate ATP via oxidative phosphorylation [76]. Acetyl‐CoA carboxylase, the key enzyme responsible for producing malonyl‐CoA, has a critical regulatory role. Hepatocyte‐specific deletion or pharmacological inhibition of acetyl‐CoA carboxylase increases TCA cycle intermediates and gluconeogenesis during the fed state by activating hepatic CPT‐1 and pyruvate carboxylase flux [10]. Malonyl‐CoA also functions as a rate‐limiting metabolite in DNL and as an allosteric inhibitor of carnitine palmitoyl transferase 1, thereby modulating mitochondrial β‐oxidation of long‐chain fatty acids [77]. Studies have reported that after Fasn knockdown, the level of malonyl‐CoA increases, and further affects the kinase activity of mTOR and downstream pathways [74].

Adipogenesis is a process in which SVF cells undergo coordinated and multifaceted changes in gene expression and protein composition as they differentiate into mature adipocytes. Although the mechanisms regulating SVF cells differentiation have been extensively studied, the processes underlying adipocyte hypertrophy and hyperplasia during adult obesity remain poorly understood. In this study, we found that the *Ddrgk1^K268R^* mutation leads to restricted adipocyte enlargement and reduced WAT mass, while the early differentiation capacity of SVF cells remains largely unaffected. Adipose tissue‐specific deletion of *Ddrgk1* reduced both adipocyte number and size, resulting in a marked decrease in WAT mass. In contrast to the severe and lethal phenotypes observed in mice lacking the causative genes *cTAGE5, Bscl2* or *Agpat2* [78], which exhibit profound WAT loss accompanied by premature mortality, adipose‐specific *Ddrgk1* knockout mice remained viable. Notably, despite reduced adiposity, these mice displayed improved glucose tolerance and enhanced insulin sensitivity, indicating that DDRGK1 plays a critical role in adipose tissue development while exerting a distinct influence on systemic metabolic homeostasis. Moreover, our data show that the *Ddrgk1^K268R^* mutation does not affect BAT or liver mass, nor does it alter the mRNA expression of transcription factors associated with BAT function. These findings suggest that *Ddrgk1^K268R^* has a limited impact on BAT formation, while its influence in the liver is mainly related to protein folding regulation rather than metabolic function.

In SVF cells cultures, DDRGK1 deficiency impairs lipid droplet formation, and this defect is only partially recovered by exogenous PA in *Ddrgk1^K268R^* cells, indicating that the point mutation specifically disrupts the DNL of palmitate. Reduced FASN protein and downstream palmitate derivatives levels trigger a compensatory feedback mechanism that increases the transcriptional expression of FASN and other lipid metabolism‐related genes, enhancing cellular lipid turnover. Consequently, *Ddrgk1^K268R^* mice fed a HFD exhibit lower body weight, reduced fat mass, improved glucose tolerance, increased energy expenditure and attenuated hepatic steatosis. These findings reveal that the *Ddrgk1^K268R^* mutation drives metabolic reprogramming toward oxidative phosphorylation by reducing FASN protein levels, leading to the accumulation of DNL intermediates such as acetyl‐CoA and malonyl‐CoA. This underscores DDRGK1's critical role in coordinating lipogenesis and energy metabolism in adipocytes.

In summary, our work defines DDRGK1 as a pivotal node linking FASN post‐translational control to systemic energy balance. By uncovering UFMylation‐mediated stabilization of FASN, identifying DDRGK1's regulation of metabolic enzymes beyond ER stress, characterizing the consequences of altered DNL intermediates, and demonstrating extensive reprogramming of adipocyte metabolic fluxes, we provide a comprehensive understanding of how DDRGK1 governs adipocyte biology. These insights suggest that targeting the DDRGK1‐UFMylation‐FASN axis could restore metabolic homeostasis in obesity and related disorders.

## 4 Methods

### 4.1 Transcriptomic Analysis Identified the Expression of FASN

We analyzed RNA‐seq data from subcutaneous adipose tissue of 770 participants in GEO dataset GSE70353, stratified by BMI into normal weight (n = 154, BMI 18.5–23.9), overweight (n = 372, BMI 24–27.9), and obese (n = 244, BMI ≥ 28) groups. Raw counts were processed using GEO2R with TMM normalization and voom transformation, followed by surrogate variable analysis for batch correction. *FASN* expression differences were assessed via one‐way ANOVA followed by Tukey's multiple comparison, while Pearson correlations evaluated associations with BMI and log‐transformed HOMA‐IR. All analyses used R v4.1.0 (limma/edgeR) with two‐sided tests (α = 0.05).

### 4.2 Ethics Statement

We analyzed omental adipose tissue samples from 23 patients (15 male and 8 female; age range: 51–87 years) undergoing abdominal surgery. The cohort included 12 normal‐weight individuals (BMI 18.5–23.9 kg/m^2^) and 11 overweight individuals (BMI 24.0–29.9 kg/m^2^), with detailed demographic characteristics summarized in Table S1. All procedures were approved by the Ethics Committee of Shanghai Ninth People's Hospital, Shanghai Jiao Tong University School of Medicine (Approval No. SH9H‐2025‐T236‐2) and were conducted in accordance with the Declaration of Helsinki. Written informed consent was obtained from all participants prior to sample collection.

### 4.3 Animal Experiments

*Ddrgk1^flox/flox^* and *Ddrgk1^K268R^* mouse lines were described in our previous study [64]. *Ddrgk1^flox/flox^* mice were crossed with *CAGG*‐CreERTM mice to generate tamoxifen‐inducible *Ddrgk1* knockout mice. Adipocyte‐specific deletion of *Ddrgk1* was generated by crossing *Ddrgk1* *^*flox/flox*^* mice with *Adipoq*‐Cre transgenic mice. All mouse lines were maintained on a C57BL/6J background and were obtained from Cyagen Biosciences. All mice were maintained under specific pathogen‐free conditions at 22°C with 50% humidity and a 12‐h light/dark cycle. All animal procedures were approved by the Institutional Animal Care Committee of Shanghai Ninth People's Hospital, Shanghai Jiao Tong University School of Medicine and conducted in accordance with NIH and institutional guidelines (Approval No. SH9H‐2025‐A1600‐1).

For the HFD time‐course study, 5‐week‐old male WT mice were randomly divided into four dietary regimens: (1) continuous chow diet (CD, 10% fat) for 8 weeks; (2) 1 week CD followed by 7 weeks HFD (60% fat); (3) 4 weeks CD followed by 4 weeks HFD; and (4) continuous HFD for 8 weeks. Body composition was analyzed at endpoint before tissue collection, with iWAT either snap‐frozen at −80°C or fixed in 4% paraformaldehyde. For inducible knockout studies, 8‐week‐old WT and *Ddrgk1^CAGG‐ERT^* mice received tamoxifen (75 mg/kg/day for 5 days) prior to HFD feeding, while another cohort of 20‐week‐old HFD‐fed mice underwent late induction. Mice were monitored weekly and euthanized upon reaching humane endpoints. In parallel, 8‐week‐old WT and *Ddrgk1^K268R^* mice were fed either CD or HFD for 15 weeks, followed by comprehensive metabolic phenotyping and tissue collection (eWAT, iWAT, BAT and liver) for molecular and histological analyses.

### 4.4 Metabolic Cage

Total energy expenditure (EE) was measured using an indirect calorimetry system (Promethion, Sable Systems) at a controlled ambient temperature of 22°C. Mice fed a standard chow diet were individually housed in metabolic cages and allowed a 24‐h acclimation period before data acquisition. Metabolic parameters were continuously recorded for 48 h. During the experiment, lighting and environmental temperature were strictly controlled to maintain normal circadian rhythms, with a light cycle of 08:00–20:00 (light) and 20:00–08:00 (dark). Adequate ventilation of the metabolic chamber was ensured, and external disturbances including daytime human activity and environmental noise were minimized. Following the calorimetry period, mice were weighed again, and remaining water intake was measured. Oxygen consumption (VO_2_) and carbon dioxide production (VCO_2_) were recorded every 5 min during the 12‐h dark phase. Subsequently, parameters including food intake, locomotor activity, energy expenditure and respiratory exchange ratio were calculated using the CalR online tool (Version 2) [79].

### 4.5 Body Composition Measurement

Total body fat and lean mass in conscious, unrestrained live mice were measured using time‐domain nuclear magnetic resonance relaxometry (MiniSpec LF50, Bruker). Animals were gently placed into a custom‐sized transparent plastic holder without sedation or anesthesia, which was then inserted into the designated tubular chamber of the MiniSpec LF50 system. To ensure measurement accuracy, mice were kept still throughout the approximately 2‐min scanning process.

### 4.6 Glucose Tolerance Test and Insulin Tolerance Test

For GTT, mice were fasted overnight for 12 h prior to intraperitoneal injection of D‐glucose (200 mg/mL in saline) at a dose of 2 g/kg body weight. Blood glucose levels were measured from tail vein samples at 0 (baseline), 15, 30, 60, 90, and 120 min post‐injection using a glucometer (Accu‐Chek). During the test, mice were randomized and housed in blinded cages to minimize bias. For ITT, mice were fasted for 6 h before receiving an intraperitoneal injection of insulin (0.75U/kg body weight). Blood glucose concentrations were measured at 0, 15, 30, 60, 90, and 120 min following insulin administration using tail vein sampling and a glucometer. Mice were similarly randomized and blinded during the procedure.

### 4.7 Blood Biochemistry

Mice were anesthetized and blood was collected via cardiac puncture. The collected blood was allowed to clot at room temperature for 30 min and then centrifuged at 3000 rpm for 20 min at 4°C. Approximately 100 µL of serum supernatant was harvested for biochemical analysis, while the remaining serum was stored at −80°C until further use. Serum levels of triglyceride, glucose, and liver and kidney function markers were measured using a biochemical analyzer (MNCHIP, Pointcare V3) according to the manufacturer's instructions.

### 4.8 Histological Analysis

Adipose and liver tissues were fixed in 4% paraformaldehyde at 4°C overnight, processed through graded ethanol dehydration, embedded in paraffin, and sectioned at 5 µm thickness. For hematoxylin and eosin (HE) staining, sections were deparaffinized with xylene, rehydrated in a graded ethanol series, stained with hematoxylin for 3 min, and counterstained with eosin for 2 min. Slides were dehydrated, cleared, and mounted with DPX for microscopic examination to assess tissue morphology.

For Oil Red O staining, frozen liver sections (10 µm) and cultured adipocytes were analyzed. Liver sections were fixed in 10% neutral‐buffered formalin for 1 h at room temperature, washed with distilled water, and pretreated with 60% isopropanol. Cultured adipocytes were fixed with 4% paraformaldehyde for 15 min, rinsed with PBS, and treated with 60% isopropanol. Both samples were stained with Oil Red O working solution (0.5% in isopropanol, diluted with water) for 10 min, rinsed in 60% isopropanol, and counterstained with hematoxylin for 1 min. Liver sections were mounted with glycerin jelly, and adipocytes were imaged directly. Lipid accumulation was quantified using ImageJ software and expressed as the percentage of the stained area relative to the total sample area.

### 4.9 Immunohistochemical Staining

Paraffin‐embedded liver and adipose tissue sections (5 µm) were deparaffinized in xylene, rehydrated through a graded ethanol series, and subjected to antigen retrieval by boiling in citrate buffer (10 mM, pH 6.0) for 15 min. After cooling to room temperature, sections were blocked with 5% bovine serum albumin in PBS for 1 h to reduce nonspecific binding. The sections were incubated overnight at 4°C with primary antibodies. After washing in PBS, sections were incubated with biotinylated secondary antibodies for 1 h at room temperature, followed by treatment with a streptavidin‐HRP complex for 30 min. Staining was developed using 3,3′‐diaminobenzidine, and sections were counterstained with hematoxylin. Stained sections were dehydrated and cleared for microscopic evaluation. All of these antibodies are provided in the Supplementary Table S3.

### 4.10 RT‐qPCR Assay

Total RNA was extracted from tissue samples and cultured cells using TRIzol reagent according to the manufacturer's instructions. For tissues, approximately 50 mg of liver or adipose tissue was homogenized in TRIzol. For cells, pellets from 1 × 10^6^ cells were lysed directly in TRIzol. The RNA concentration and purity were determined using a NanoDrop spectrophotometer. RNA (1 µg) was reverse‐transcribed into cDNA using the PrimeScript RT reagent kit. Quantitative PCR was performed using SYBR Green Master Mix on a QuantStudio 5 Real‐Time PCR System. Gene‐specific primers were designed to amplify target genes and normalized to the housekeeping gene 36B4 or β‐actin. The relative expression levels were calculated using the ΔΔCt method. Primer sequences for the genes of interest are provided in the Supplementary Table S4.

### 4.11 Western Blot

For tissues, approximately 50 mg of liver or adipose tissue was homogenized in RIPA buffer supplemented with protease and phosphatase inhibitors using a tissue homogenizer. For cells, 1 × 10^6^ cells were lysed directly in ice‐cold RIPA buffer. Lysates were incubated on ice for 30 min and centrifuged at 12 000 × g for 15 min at 4°C to remove debris. The supernatants were collected, and protein concentrations were determined using a BCA Protein Assay Kit. Equal amounts of protein (20–30 µg) were mixed with 5× SDS loading buffer, boiled at 95°C for 5 min and separated by SDS‐PAGE. Proteins were transferred onto PVDF membranes and blocked in 5% nonfat milk in TBST for 1 h at room temperature. The membranes were incubated overnight at 4°C with primary antibodies. After washing, membranes were incubated with HRP‐conjugated secondary antibodies for 1 h at room temperature. Proteins were visualized using an enhanced chemiluminescence system and imaged with a touch imager XLi. Band intensities were normalized to β‐actin.

### 4.12 Immunofluorescence Staining

Liver and adipose tissue sections (5 µm) or cultured cells grown on glass coverslips were used for immunofluorescence staining. Paraffin‐embedded tissue sections were deparaffinized in xylene, rehydrated through graded ethanol, and subjected to antigen retrieval in citrate buffer (10 mM, pH 6.0) at 95°C for 15 min. Cultured cells were fixed with 4% paraformaldehyde for 15 min at room temperature, washed with PBS, and permeabilized with 0.1% Triton X‐100 for 10 min.

Both tissue sections and cells were blocked with 5% bovine serum albumin in PBS for 1 h at room temperature to prevent nonspecific binding. Samples were incubated overnight at 4°C with primary antibodies. After washing with PBS, samples were incubated with fluorescently labeled secondary antibodies for 1 h at room temperature in the dark. For nuclear counterstaining, DAPI was applied for 5 min. Tissue sections were mounted with fluorescence mounting medium. Fluorescence images were captured using a confocal microscope (Zeiss LSM880). Fluorescence colocalization was quantified using ImageJ's Plot Profile tool, following established protocols. Intensity profiles along defined lines through regions of interest were used to assess overlap between signals, as previously described in Zhou et al. [80].

### 4.13 Immunoprecipitation and Mass Spectrometry

HEK293T cells were transfected with either empty FLAG vector or FLAG‐tagged FASN plasmids using Lipofectamine 3000 according to the manufacturer's instructions. After 48 h, cells (1 × 10^7^) were harvested and lysed in ice‐cold IP lysis buffer supplemented with protease and phosphatase inhibitors. Lysates were centrifuged at 12 000 × g for 15 min at 4°C to remove debris. Supernatants containing 2 mg total protein were incubated with anti‐DYKDDDDK tag immunomagnetic beads overnight at 4°C with gentle rotation. Beads were washed five times with lysis buffer to remove nonspecific proteins. Peptides were dissolved in a gradient manner from Bound proteins. The peptide segments separated by liquid phase were ionized by the nanoESI source and then entered the tandem mass spectrometer Orbitrap Fusion Lumos (Thermo Fisher Scientific, San Jose, CA) for Data Dependent Acquisition pattern detection. Protein spectrum identification mainly involves matching experimental tandem mass spectrometry data with theoretical mass spectrometry data simulated in the database to obtain protein identification results. The identified differential proteins have been presented in the Supplementary Table S5.

### 4.14 RNA Sequencing

Total RNA was extracted from tissue samples (50 mg of liver and adipose tissue) or cultured cells (1 × 10^6^ cells) using TRIzol reagent according to the manufacturer's protocol. total RNA was treated by mRNA enrichment method. The obtained RNA was fragmented by breaking buffer. Reverse transcription was performed with random N6 primers, and then cDNA two strands were synthesized to form double‐stranded DNA. The ends of the synthesized double‐stranded DNA are flattened and phosphorylated at the 5' end, forming A sticky end with a protruding “A” at the 3' end, and then connected to a bubble‐shaped junction with a protruding “T” at the 3' end. The ligation products were amplified by PCR using specific primers. The PCR products were thermally denatured into single strands, and then a bridge primer was used to cyclize the single‐stranded DNA to obtain a single‐stranded circular DNA library. The constructed library is subject to quality inspection and sequencing after passing the inspection. Sequencing was conducted using the DNBSEQ platform and PE150 (read length) sequencing. The data obtained from sequencing is called raw reads or raw data. Subsequently, quality control is conducted on the raw reads to determine whether the sequencing data is suitable for subsequent analysis. After quality control, the clean reads obtained through filtering are aligned to the reference sequence. After the alignment is completed, by statistically analyzing the alignment rate, the distribution of reads on the reference sequence, etc., it is determined whether the alignment result passes the second quality control. If passed, quantitative gene analysis and various analyses based on gene expression levels (principal components, correlations, differential gene screening, etc.) will be carried out. For the differentially expressed genes among the screened samples, GO functional significance enrichment analysis, pathway significance enrichment analysis, clustering, protein interaction networks and transcription factors and other more in‐depth mining analyses will be conducted.

### 4.15 Proteomics

Frozen iWAT samples (50 mg, n = 3) were homogenized in liquid nitrogen and lysed in ethanol (1 mL) with ultrasonication (5 min, ice) and vortexing (5 min, RT). After centrifugation (15 000 g, 4°C, 10 min), pellets were resuspended in urea buffer (8 M urea, 1 mM PMSF, 2 mM EDTA), sonicated and centrifuged. Protein concentration was determined by BCA assay. For digestion, aliquots (100 µg) were reduced with 5 mM DTT (37°C, 45 min), alkylated with 11 mM iodoacetamide (RT, dark, 15 min), and digested with trypsin (Promega, 1:50 w/w, 37°C, 16 h) in 25 mM ammonium bicarbonate. Peptides were acidified with TFA, desalted using C18 (Millipore, Billerica, MA) resin and determine the peptide concentration using a Pierce Quantitative Peptide Assay Kit with standards (Thermo Fisher). Liquid chromatography (LC) separation was performed on a nanoElute UHPLC (Bruker) with Aurora C18 column (25 cm × 75 µm, 1.6 µm; IonOpticks) at 50°C using a 40‐min gradient (0.3 µL/min) of 0.1% formic acid in water/ACN. MS analysis used a timsTOF Pro2 (Bruker) in diaPASEF mode (m/z 100–1700, ion mobility 0.85–1.3 Vs/cm^2^) with collision energy ramping (27–45 eV). Data were processed with DIA‐NN v1.8.1 against UniProt Mouse proteome (UP000000589) using library‐free prediction and MBR, with FDR <1% at both peptide and protein levels (median CV <15%, >4000 proteins/sample).

### 4.16 Single‐Nucleus RNA Sequencing

Fresh adipose tissue samples were processed to isolate nuclei by mechanical homogenization followed by filtration through a 40 µm strainer. Nuclei were purified using a sucrose gradient and stained with DAPI to assess integrity and viability, achieving >85% viable nuclei. Single‐nucleus libraries were prepared using the Chromium Single Cell 3′ Library & Gel Bead Kit v3 (10× Genomics) according to the manufacturer's instructions. Approximately 10 000 nuclei were loaded per channel to generate Gel Bead‐In Emulsions (GEMs). Libraries were sequenced on an Illumina NovaSeq 6000 platform generating paired‐end reads (28 bp and 91 bp). Raw data were processed using Cell Ranger with alignment to the mouse reference genome (GRCm39). Downstream analysis was performed with Seurat (v4.0), including filtering out nuclei with high mitochondrial gene content (>10%) or low gene counts (<200), normalization, dimensionality reduction, clustering, and annotation using canonical marker genes.

Metabolic flux was inferred from snRNA‐seq data using the scFEA algorithm, which estimates cell‐type–specific metabolic activity based on the expression levels of catalytic enzymes. scFEA is built on the core assumption that the overall flux of a metabolic module is primarily constrained by the transcript abundance of its key rate‐limiting enzymes. To model this, scFEA consolidates metabolic reactions into 140 predefined metabolic modules. For each metabolic module i, the flux (F_i_) is modeled as a weighted linear combination of the expression levels (E_j_) of its associated genes j:

$$F_{i}=\Sigma \left(w_{ij}\times E_{j}\right)+B_{i}$$

where w_ij_ represents the contribution of gene j to the flux of module i, and b_i_ is a module‐specific bias term. Importantly, these weights are not pre‐assigned. Instead, scFEA learns w_ij_ and b_i_ from the data through a multilayer perceptron model, enabling data‐driven reconstruction of relative metabolic flux across single‐nucleus transcriptomes.

### 4.17 Quantitative Lipidomics

iWAT samples were thoroughly minced and homogenized. Multiple subsamples were collected, and approximately 10 mg (±1 mg) of tissue was weighed into pre‐labeled centrifuge tubes. Lipid extraction was performed by adding 1 mL of extraction solvent containing internal standards (methyl tert‐butyl ether:methanol = 3:1, v/v), followed by vortexing for 15 min. After adding 200 µL of ultrapure water, samples were shaken for 1 min and allowed to stand at 4°C for 10 min to facilitate phase separation. The mixtures were centrifuged at 12 000 rpm for 1 min at 4°C, and 200 µL of the upper organic phase was transferred to clean 1.5‐mL tubes and dried completely at 20°C. The dried residues were reconstituted in 400 µL of resuspension solvent (acetonitrile:isopropanol = 1:1, v/v), vortexed for 3 min, centrifuged at 12 000 rpm for 3 min, and the supernatants were collected for LC–MS/MS analysis.

Lipidomic profiling was performed using a UPLC system (ExionLC AD, SCIEX) coupled to a QTRAP 6500+ LC‐MS/MS system (SCIEX, USA) equipped with an ESI Turbo Ion‐Spray source operating in both positive and negative ion modes. Chromatographic separation was achieved on a Thermo Accucore C30 column (2.6 µm, 2.1 × 100 mm) using a linear gradient of acetonitrile/isopropanol. Source parameters were set as curtain gas 35 psi, GS1 45 psi, GS2 55 psi, temperature 500°C, and ion spray voltages of +5500 V/−4500 V.

Lipid identification was performed using the in‐house MetWare Database (MWDB) based on retention time, precursor ions, and characteristic fragment ions. Mass spectrometry data were processed using Analyst 1.6.3, and quantitative values were derived from peak area ratios normalized to internal standards and calibration curves. Raw lipid data were subsequently processed using R‐based packages for statistical analysis.

Because FDR filtering was not applied in this workflow, differential lipid species were determined using two criteria: Variable Importance in Projection (VIP) greater than 1 from OPLS‐DA, reflecting contribution to group separation, and Student's t‐test p < 0.05, indicating statistical significance. Lipid species passing these criteria were retained for downstream biological interpretation and pathway analysis.

### 4.18 Preadipocyte Isolation and Adipogenic Differentiation In Vitro

After the above‐mentioned model mice were euthanized, the iWAT mice were dissected and weighed. Some primary mouse SVF was isolated from iWAT, digested with three thousandths of collagenase I, digested at 37°C for 45 min, centrifuged at 500 g for 5 min, and the digestion was terminated by adding α‐DMEM containing 10% bovine serum. Then, the undigested tissue was removed by centrifugation, and SVF pre‐adipocytes were obtained in the pellets. The newly isolated SVF cells were sown and cultured in a growth medium containing α‐DMEM, 10% FBS and 1% penicillin/streptomycin at 37°C and 5% CO_2_ until 100% fusion was achieved. For adipogenic differentiation, mouse adipose‐derived mesenchymal stem cells (OriCell, MUXMX‐90031) were used to replace the growth medium, and the cycle was carried out with solution A for 3 days and solution B for 1 day until obvious lipid droplets appeared.

### 4.19 Culture of HEK293T Cells and Plasmid Transfection

HEK‐293 T are immortalized human renal epithelial cell lines cultured in DMEM (5% FBS and 1% penicillin/streptomycin) (American Type Culture Collection, ATCC; # CRL‐3216). 1 day prior to transfection, cells were trypsinized, counted, and seeded at an appropriate density in six‐well plates. When cells reached 70%–90% confluence, the culture medium was replaced with serum‐free DMEM. Transfection was performed using Lipofectamine 3000 according to the manufacturer's instructions, with 2 µg plasmid DNA per well. 6 h post‐transfection, the medium was replaced with complete growth medium. Cells were harvested 48 h after transfection for subsequent analyses. Plasmids used in this study, including Flag‐FASN, Myc‐DDRGK1, Myc‐K268R, Myc‐UFL1, Myc‐UFC1, HA‐UFM1, and Myc‐UB were purchased from Tsingke Biotech Co., Ltd.

### 4.20 Co‐Immunoprecipitation and Endogenous Immunoprecipitation

HEK293T cells or tissue samples (human omental adipose tissue or mouse iWAT for endogenous IP) were lysed in ice‐cold IP lysis buffer supplemented with protease and phosphatase inhibitors. Lysates were incubated on ice for 30 min and centrifuged at 12 000 × g for 15 min at 4°C to remove debris.

For Co‐IP in HEK293T cells, equal amounts of protein (1–2 mg) were incubated overnight at 4°C with pre‐conjugated antibody magnetic beads under gentle rotation. For endogenous IP, magnetic beads were first incubated with FASN antibody for 15 min to allow antibody conjugation, followed by addition of prepared tissue lysates and incubation overnight at 4°C. Beads were washed five times with lysis buffer to minimize nonspecific binding. Bound proteins were eluted by adding 5× SDS loading buffer and boiling to denature the proteins, followed by analysis via SDS‐PAGE and western blotting using the corresponding antibodies.

### 4.21 UFM1 Modification Assay and Ubiquitylation Modification Assay

HEK293T cells were transfected with plasmids encoding target proteins along with HA‐tagged UFM1 or HA‐tagged ubiquitin, as indicated. After 48 h, cells were lysed in denaturing buffer and boiled for 10 min to disrupt non‐covalent interactions. Lysates were then diluted tenfold with IP buffer supplemented with protease inhibitors. Target proteins were immunoprecipitated using specific antibodies conjugated to magnetic beads overnight at 4°C. Beads were washed five times with IP buffer, and bound proteins were eluted by boiling in SDS loading buffer. Samples were subjected to SDS‐PAGE and immunoblotted with antibodies to detect UFM1 or ubiquitin conjugates.

### 4.22 *Ddrgk1* Knockout in SVF Cells

SVF cells were isolated from *Ddrgk1^CAGG‐ERT^* mice as previously described. To induce *Ddrgk1* knockout, cells were treated with tamoxifen. Knockout efficiency was confirmed by western blot analysis.

### 4.23 Cell Viability Test

Cell viability of SVF cells treated with tamoxifen and PA was assessed using the cell counting kit‐8. SVF cells were seeded into 96‐well plates at a density of 3000 cells per well. After 12 h, tamoxifen or PA was added to the wells, and cells were incubated for 24 or 48 h at 37°C. At the end of treatment, 100 µL of fresh culture medium containing 10 µL of CCK‐8 reagent was added to each well and incubated for 1.5 h at 37°C. Wells without cells but containing CCK‐8 reagent served as blanks, while untreated cells served as controls. Absorbance at 450 nm was measured using a microplate reader (Tecan Group, Infinite M200 PRO). Cell viability was calculated based on the optical density values.

### 4.24 Seahorse Assay

SVF cells isolated from mice were seeded into XF‐96 cell culture plates (Seahorse Bioscience, USA) at 5 × 10^3^ cells per well and cultured for 24 h. Cells were then induced to differentiate with adipogenic induction medium for 4 days. For mitochondrial respiration, the oxygen consumption rate (OCR) was measured using the Seahorse XF Cell Mito Stress Test with sequential injections of 2 µM oligomycin, 2 µM FCCP and 0.5 µM rotenone/antimycin. Fatty acid oxidation (FAO) was assessed by Seahorse XF FAO assay, with sequential injections of 4 µM etomoxir, 2 µM oligomycin, 2 µM FCCP and 0.5 µM rotenone. For glycolysis, the extracellular acidification rate (ECAR) was measured using the Seahorse XF Glycolytic Rate Assay, with sequential injections of 10 mM glucose, 2.5 µM oligomycin and 50 mM 2‐deoxy‐D‐glucose (2‐DG). All experiments were performed according to the manufacturer's instructions, and data were analyzed using Wave software (Agilent Technologies).

### 4.25 BODIPY Staining

To observe lipid droplets, the extracted SVF cells were inoculated onto confocal cells and adipogenic induction was carried out according to the above method. After induction for 4 days, the cells were washed twice with PBS and fixed with 4% paraformaldehyde at room temperature for 15 min. The fixed cells were washed three times with PBS to remove the residual paraformaldehyde. Then, 2 µM BODIPY 493/503 (Invertrogen, D3922) diluted with PBS was incubated in the dark at room temperature for 10 min to stain neutral lipids. After staining, wash the cells three times with PBS to remove the excess dye. The cell nuclei were counterstained with 1 µg/mL DAPI in PBS for 10 min. Images were obtained using a confocal laser scanning microscope, and the fluorescence intensity and lipid droplet quantification were analyzed using ImageJ software.

### 4.26 MitoSOX Red Staining

SVF cells were differentiated into adipocytes, and on day 4 of adipogenic induction, cells were incubated with MitoSOX Red, MitoTracker Green and Hoechst 33342 for 20 min at 37°C according to the manufacturers’ protocols. Following staining and PBS washes, cells were imaged using a fluorescence microscope. Nuclear DNA was visualized with Hoechst (blue), mitochondria were labeled with MitoTracker Green (green), and mitochondrial reactive oxygen species were detected by MitoSOX Red (red).

### 4.27 Biochemical Assay and Enzyme‐Linked Immunosorbent Assay

Blood was collected via cardiac puncture from anesthetized mice and processed to obtain serum (30 min clotting at room temperature followed by centrifugation at 3000 × g for 20 min at 4°C). Stromal vascular fraction (SVF) cells were isolated from iWAT and cultured to passage 2 before analysis. Serum pyruvate and triglyceride levels were measured using commercial assay kits (Sangon Biotech) according to manufacturer protocols. Similarly, SVF cell lysates were analyzed for intracellular pyruvate and triglyceride content. Acetyl‐CoA and malonyl‐CoA concentrations in both serum and SVF cells were quantified using competitive ELISA kits (Coibo Biotech), with all assays performed in technical duplicates and including appropriate standards and controls.

### 4.28 Quantification of FASN Activity

FASN enzymatic activity in mouse iWAT was measured using a FASN Activity Assay Kit (BC0550, Solarbio) following the manufacturer's instructions. Briefly, iWAT samples were collected, mechanically homogenized in assay buffer and centrifuged to remove debris. The resulting supernatants were then used to determine FASN activity according to the kit protocol.

### 4.29 ATP Production Assay

Intracellular ATP levels were measured in differentiated SVF cells using The ATP Determination Kit (A22066, Invitrogen) according to the manufacturer's instructions. Briefly, differentiated cells were lysed, and the luminescent signal corresponding to ATP content was quantified using a microplate reader. Data were normalized to total protein concentration.

### 4.30 Statistical Analysis

All experiments were performed with at least three biological replicates, with data presented as mean ± standard deviation (SD). Statistical analyses were conducted using SPSS (v19.0, IBM). For two‐group comparisons, two‐tailed unpaired Student's *t*‐tests were used with Welch's correction for unequal variances when appropriate. Multiple group comparisons were analyzed by one‐way ANOVA with Tukey's post‐hoc test for normally distributed data, or Kruskal‐Wallis test with Dunn's correction for non‐parametric data. Statistical significance was set at *p* < 0.05, with exact *p*‐values reported unless <0.0001. All statistical tests were two‐sided, and no data points were excluded unless technical failures were identified during quality control.

## Author Contributions

Y.L. and T.Z. performed experiments, data analysis, and prepared the manuscript. X.Y., K.R., and X.C. assisted in data interpretation and manuscript refinement. L.S. and X.W. performed western blot, immunohistochemical staining, and ELISA experiments. H.W., L.C., and K.L. conducted metabolomics and lipidomic analyses. T.X., H.Z., and C.Z. performed seahorse assays and related analyses. T.G. conducted snRNA‐seq analysis and bioinformatics interpretation. P.M., A.Q., and J.Z. conceived and supervised the project, contributed to data interpretation, and prepared the manuscript. All authors provided intellectual input and reviewed the final manuscript.

## Conflicts of Interest

The authors declare no conflict of interest.

## Supporting information




**Supporting File**: advs74229‐sup‐0001‐SuppMat.docx.

## Data Availability

Refer to Supplementary Information for the patients’ information (Supplementary Table S1), key reagents (Supplementary Table S2), antibodies (Supplementary Table S3), primer sequences for RT‐qPCR (Supplementary Table S4), IP‐MS dataset (Supplementary Table S5), Metabolic cage data (Supplementary Table S6), ANCOVA results (Supplementary Table S7) and Proteomics data of iWAT (Supplementary Table S8) mentioned in the present study.
