# Supplementary material for: Ufmylation‐Deficient DDRGK1 Ameliorates Obesity by Inhibiting FASN‐Mediated Adipocyte Lipogenesis
Source: Adv Sci (Weinh). 2026 Feb 11;13(21):e14702. doi: 10.1002/advs.202514702 (PMC13073236; doi:10.1002/advs.202514702)
Supplement: Supplementary file 1 — Supporting File: advs74229‐sup‐0001‐SuppMat.docx. [file ADVS-13-e14702-s001.docx]

**Ufmylation-deficient DDRGK1 Ameliorates Obesity by Inhibiting FASN-mediated Adipocyte Lipogenesis**

Yin Li^1,2^, Tangjun Zhou^1,2^, Xiao Yang^1,2^, Kewei Rong^1^, Xiankun Cao^1^, Lei Shi^1^, Xin Wang^1^, Hongjin Wan^1^, Lei Cui^1^, Kexin Liu^1^, Tong Xing^1^, Hang Zhang^1^, Chen Zhao^1^, Tingxian Guo^1^, Peixiang Ma^1*^, Jie Zhao^1*^ & An Qin^1*^

^1^Shanghai Key Laboratory of Orthopedic Implants, Department of Orthopedics, Ninth People’s Hospital, Shanghai Jiao Tong University School of Medicine, 639 Zhizaoju Road, Shanghai, 200011, China. ^2^These authors contributed equally: Yin Li, Tangjun Zhou and Xiao Yang.

*Correspondence:

An Qin: dr_qinan@shsmu.edu.cn

Jie Zhao: profzhaojie@126.com

Peixiang Ma: mapx@shsmu.edu.cn

**
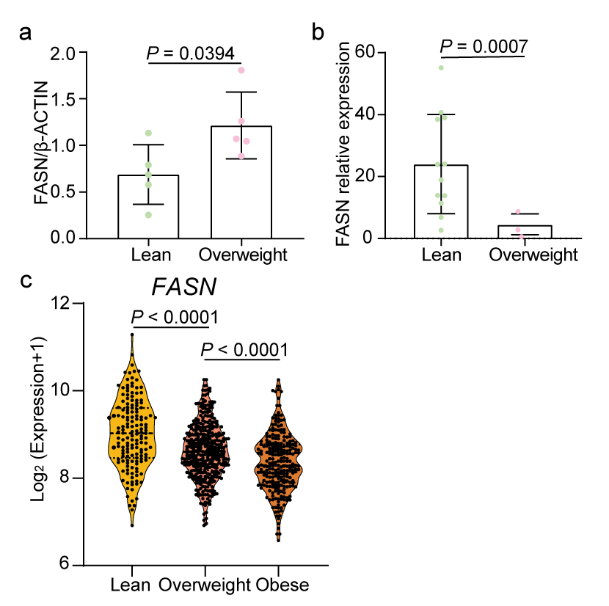
**

**Extended Data Fig. S1 |** FASN protein and gene expression in humans and mice. (a) Quantification of FASN protein levels normalized to β-actin. (b) RT‑qPCR analysis of *FASN* mRNA in human adipose tissue samples. (c) *FASN* transcript levels stratified by BMI categories. Panels (a, b): Two-tailed unpaired Student’s t-tests. Panel (c): One-way ANOVA followed by Tukey’s multiple comparison.


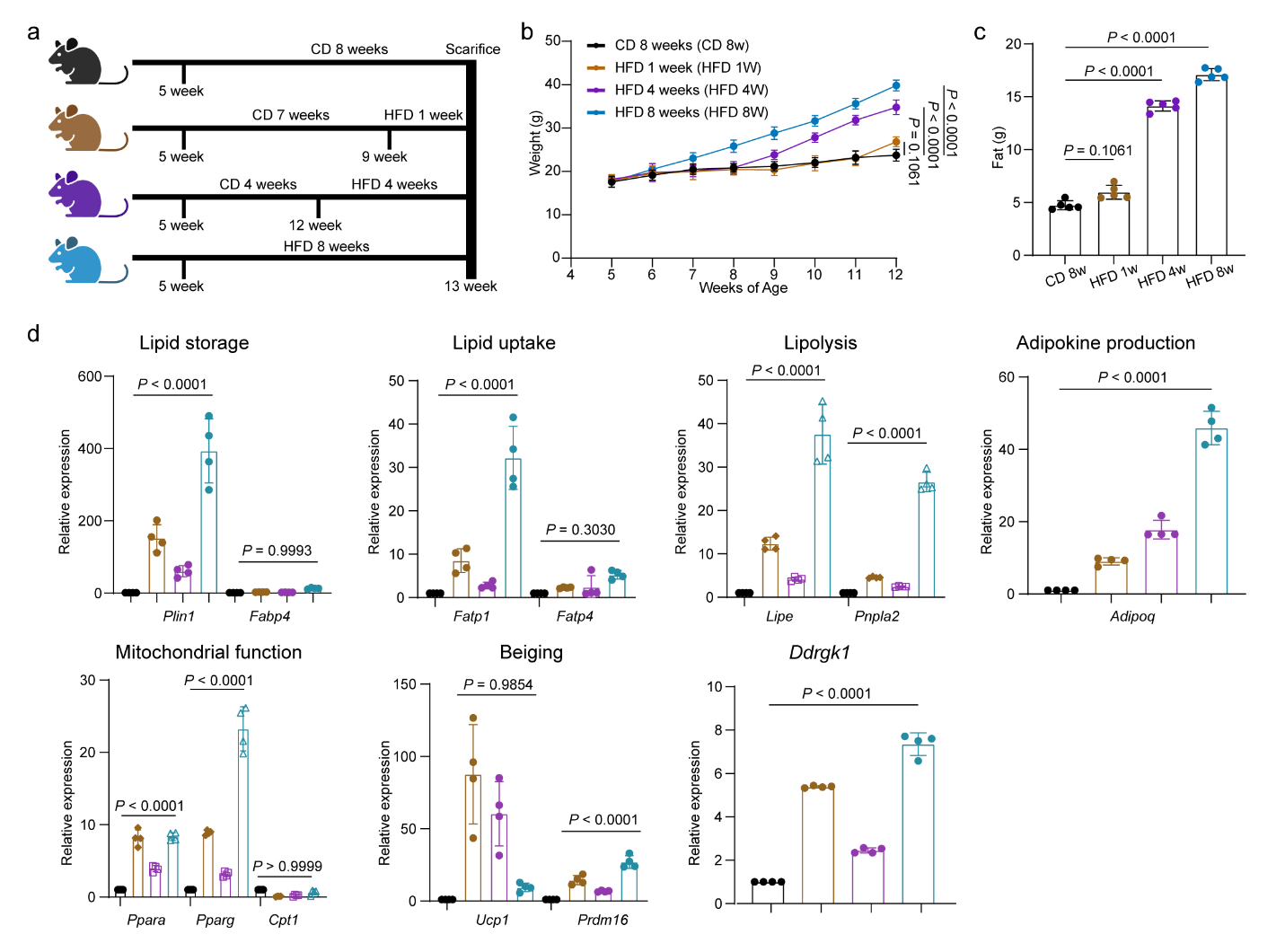


**Extended Data Fig. S2 |** Metabolic phenotype of HFD-fed mice at 12 weeks. (a-c) Longitudinal analysis. (a) Body weight progression from weeks 4-12 (arrows indicate diet switch time points). (b) Terminal body weight at week 12. (c) Fat mass quantification. Groups: n = 5 WT CD (chow diet), n = 5 WT HFD 1w (1 week HFD), n = 5 WT HFD 4w (4 weeks HFD), n = 5 WT HFD 8w (8 weeks HFD). (d) Gene expression profiling in iWAT by RT-qPCR. Functional categories: lipid storage *(Plin1, Fabp4*), uptake (*Fatp1, Fatp4*), lipolysis (*Lipe, Pnpla2*), adipokines (*Adipoq*), mitochondrial function (*Ppara, Pparg, Cpt1b*), beiging (*Ucp1, Prdm16*) and *Ddrgk1*. Data normalized to *36B4* and presented as fold-change vs CD (mean ± SEM, n = 4 biological replicates with 2 technical replicates each). Panels (b, d): One-way ANOVA followed by Tukey’s multiple comparison.


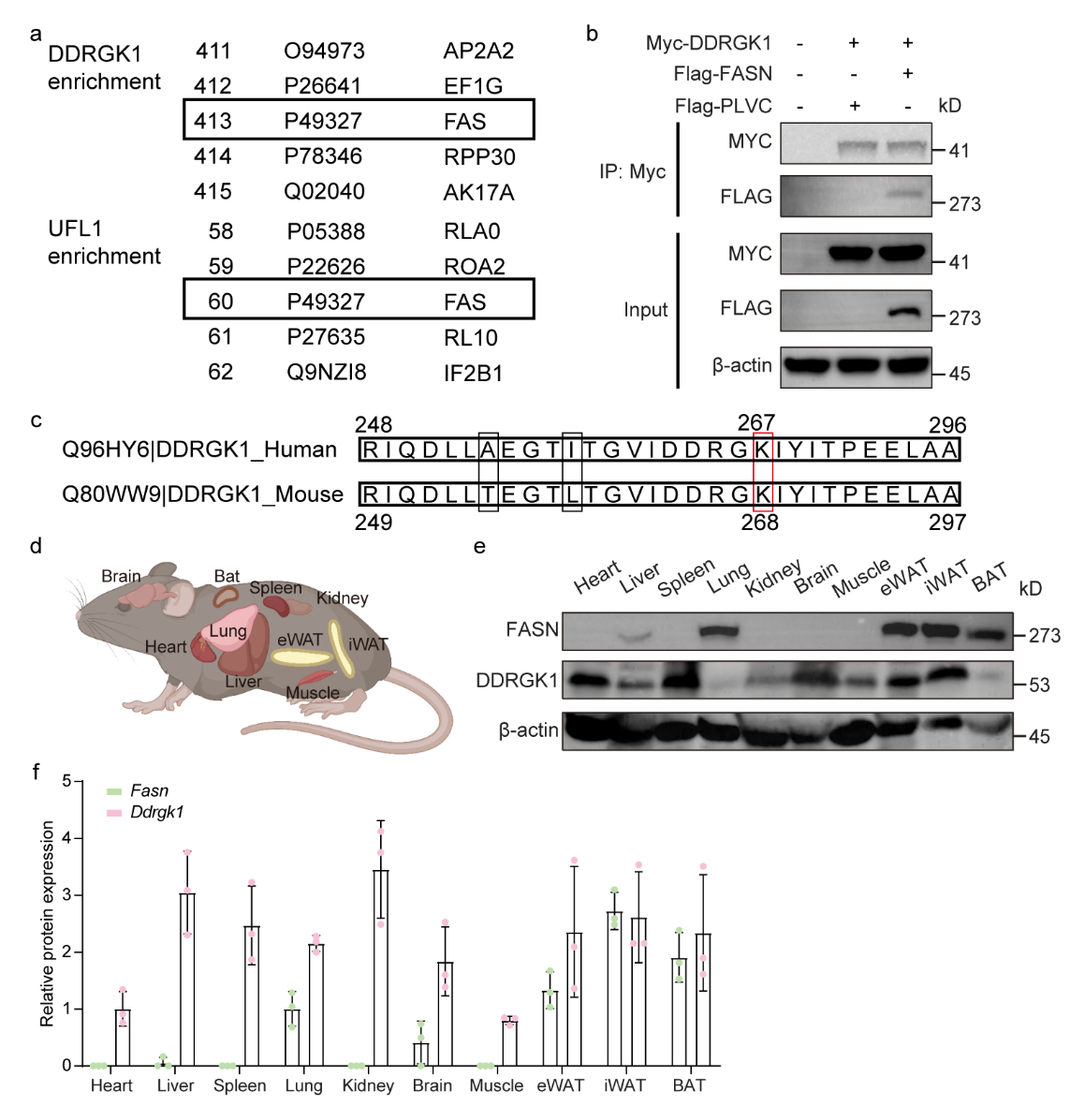


**Extended Data Fig. S3 |** Expression profiling of DDRGK1 and FASN in mouse tissues. (a) Mass spectrometry analysis of DDRGK1 and UFL1 protein enrichment^44^. (b) Validation of FASN-DDRGK1 interaction by Co-IP. (c) Cross-species sequence alignment of DDRGK1 between human (Q96HY6) and mouse (Q80WW9). Differential base pairs marked by black arrow. (d-f) Tissue distribution analysis. (e) Representative western blot of FASN and DDRGK1 in [e.g., iWAT, eWAT, liver, muscle, heart] (n = 3 mice). β-actin loading control shown. (f) Corresponding mRNA levels by RT-qPCR (normalized to *36B4*, n = 3 mice/tissue).


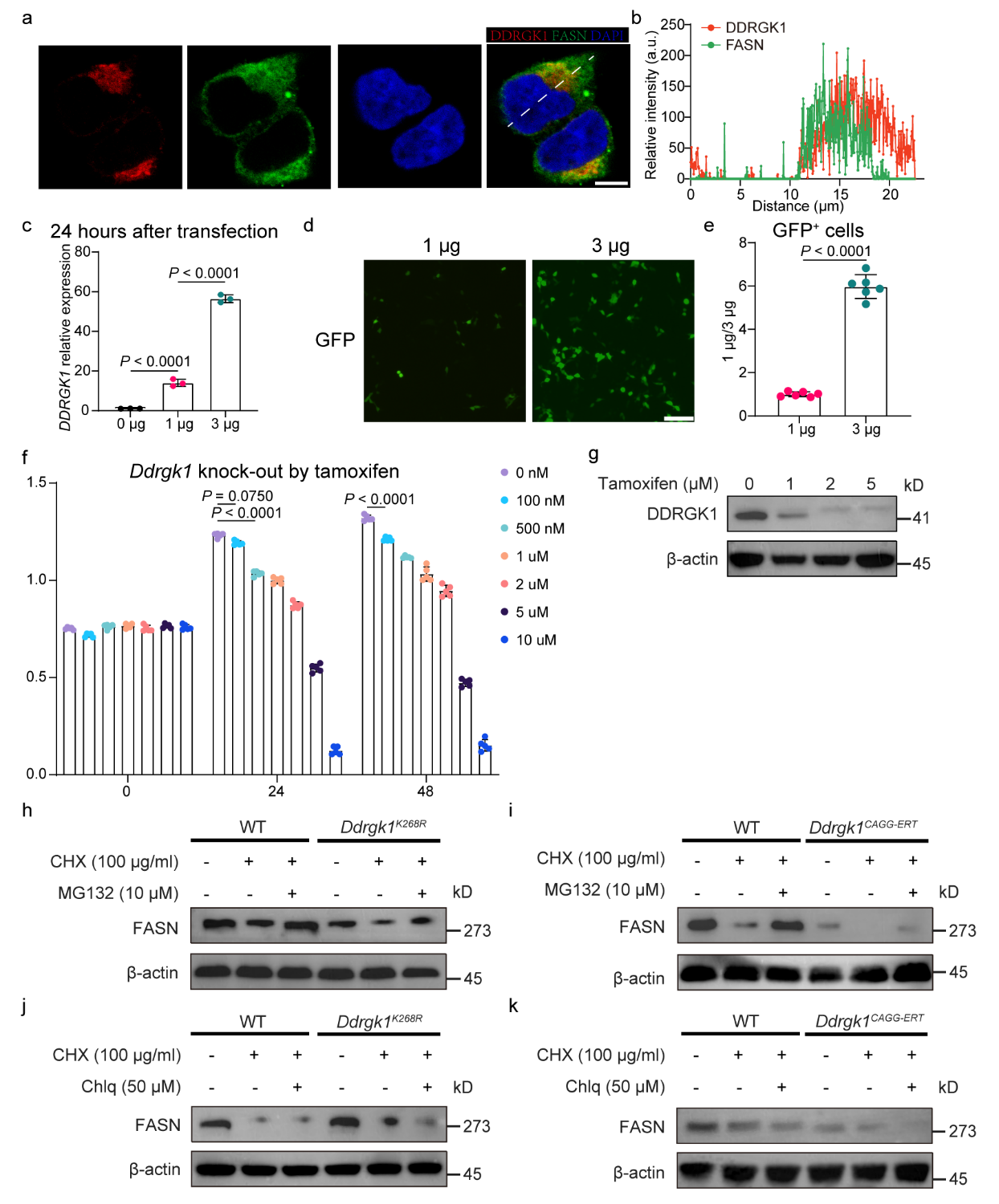


**Extended Data Fig. S4 |** Characterization of DDRGK1 localization, expression and protein stability. (a) Co-localization of endogenous DDRGK1 (red) and FASN (green) in HEK293T cells. Nuclei were counterstained with DAPI (blue) (Scale bars, 5 μm). (b) Pixel intensity plots along the white line from merged images. (c) RT-qPCR analysis of *Ddrgk1* in HEK293T cells 24 h after transfection with 0, 1, or 4 μg of MYC-GFP-DDRGK1 plasmid. (d) Representative fluorescence images of HEK293T cells transfected with 1 or 3 μg MYC-GFP-DDRGK1 plasmid for 24 h (Scale bars, 100 μm). (e) Cell viability assessment by CCK-8 assay in SVF cells isolated from *Ddrgk1^CAGG-ERT^* mice after tamoxifen treatment. Data are normalized to vehicle control (n = 5 independent isolations). (f) *Ddrgk1* knockout efficiency in tamoxifen-treated SVF cells determined by Western blot. (g-j) Protein stability assays in differentiated adipocytes. (g, h) WT and *Ddrgk1^K268R^* cells treated with cycloheximide (CHX, 100 μg/mL, 8 h), MG132 (10 μM, 8 h) or chloroquine (50 μM, 8 h) to block proteasomal or lysosomal degradation. (i, j) Parallel experiments in WT and *Ddrgk1^CAGG-ERT^* cells following tamoxifen-induced knockout. Panels (c, f): One-way ANOVA followed by Tukey’s multiple comparison. Panel (e): Two-tailed unpaired Student’s t-tests.


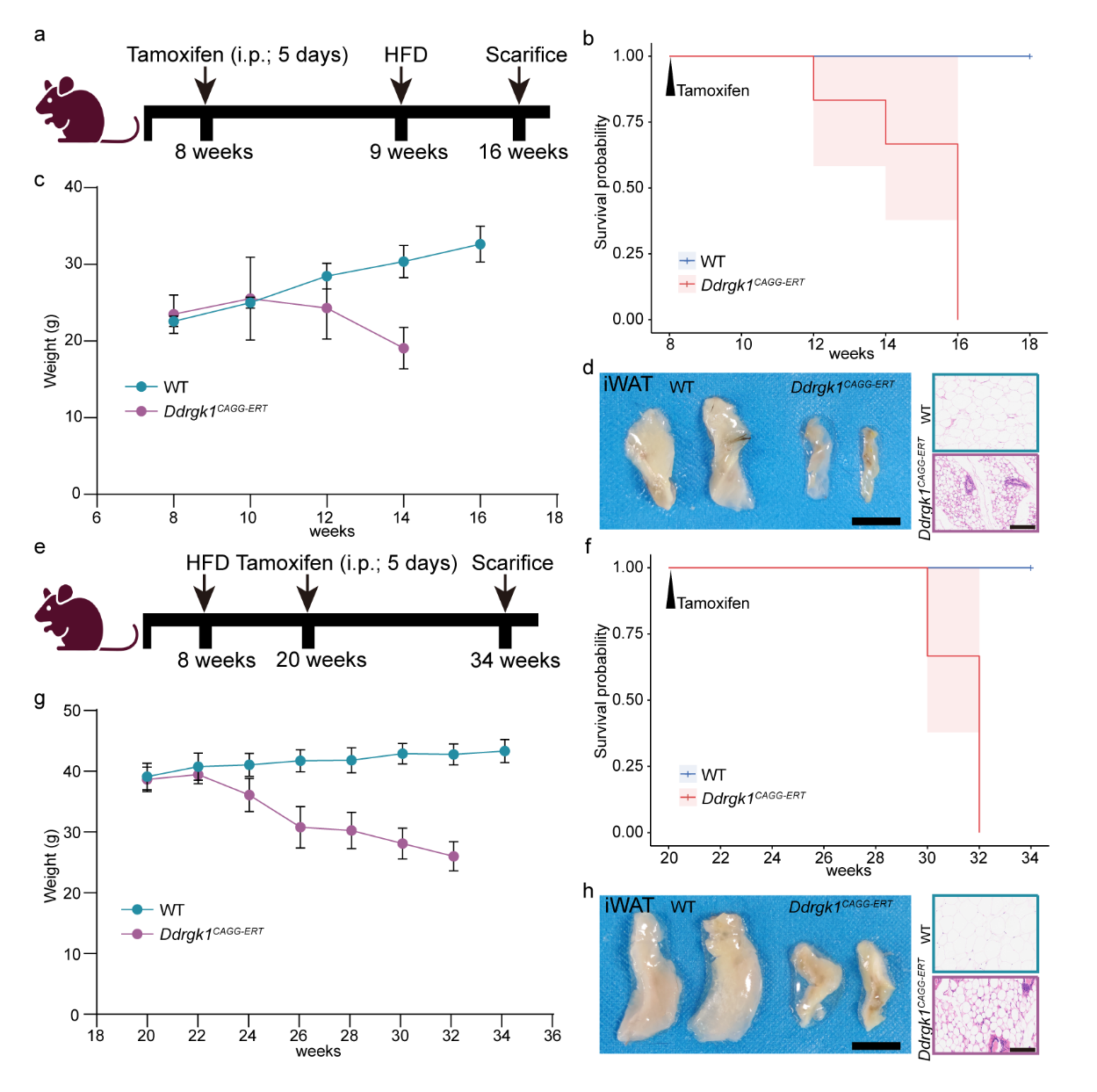


**Extended Data Fig. S5 |** Phenotypic characterization of tamoxifen-induced *Ddrgk1* knockout mice under normal and HFD conditions. (a) Schematic diagram of tamoxifen injection and subsequent HFD in mouse. (b) The survival curve of *Ddrgk1^CAGG-ERT^* mice after tamoxifen injection (75 mg/kg, i.p. 5 days). (c) The weight curve of mice on a HFD after tamoxifen injection. (d) Gross fat images of mice on a HFD after tamoxifen injection (Scale bars, 1 cm). (e) Schematic diagram of tamoxifen injection in mice on HFD. (f) Survival curve of mice on a HFD after tamoxifen injection (75 mg/kg, i.p. 5 days). (g) The weight change curve of mice on a HFD after tamoxifen injection. (h) Gross image of fat injected with tamoxifen in mice on HFD (Scale bars, 1 cm).


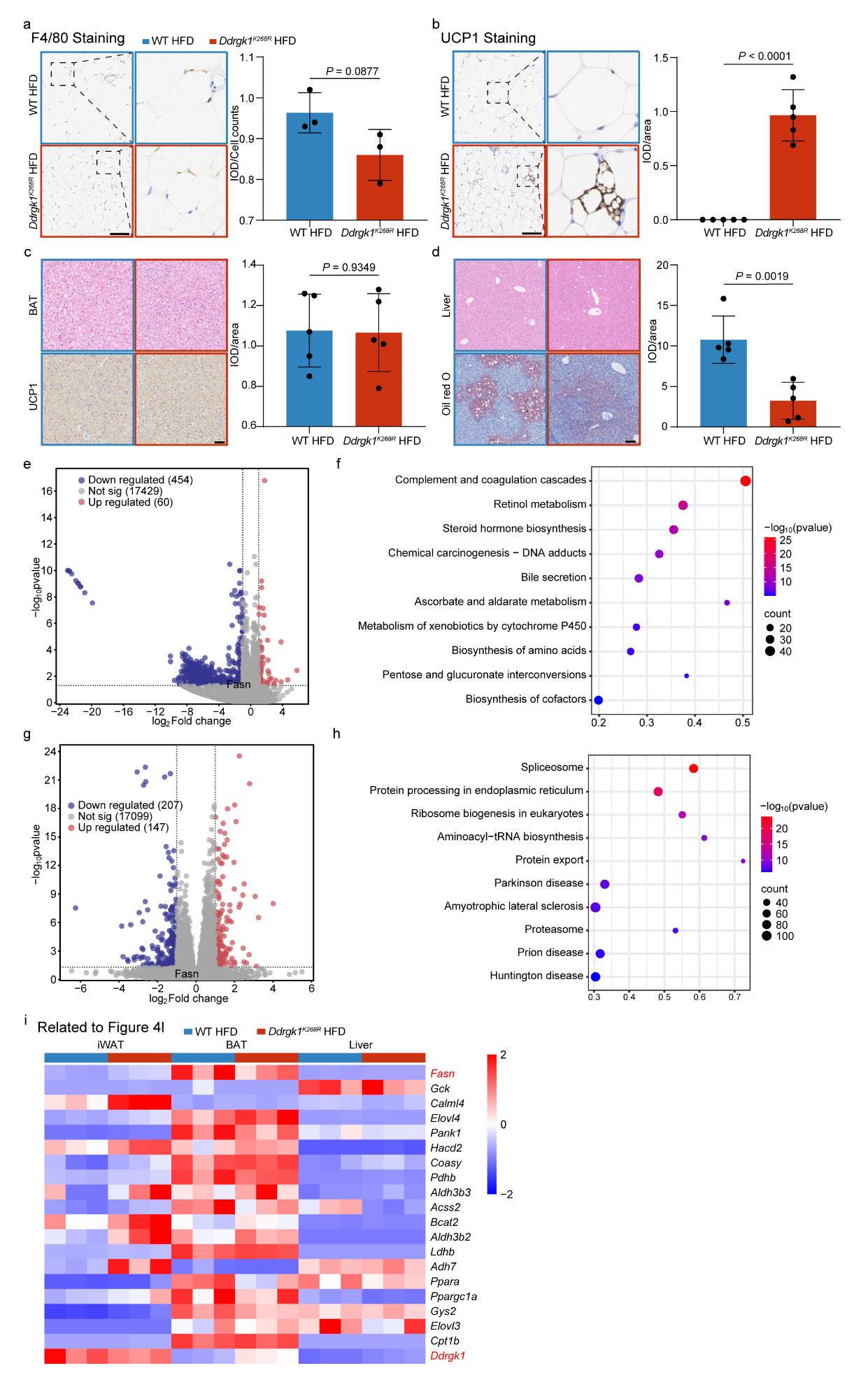


**Extended Data Fig. S6 |** Metabolic tissue characterization in HFD-fed *Ddrgk1^K268R^* mice. (a) Immunofluorescence staining of crown-like structures (F4/80+ macrophages) in iWAT sections (Scale bars, 100 μm). (b) UCP1 immunohistochemistry of iWAT demonstrating beige adipocyte formation. (c) BAT morphology and function. Top: H&E staining showing adipocyte size distribution. Bottom: UCP1 protein expression (brown DAB staining) (Scale bars, 100 μm). (d) Hepatic steatosis analysis. Top: H&E staining revealing lipid droplet accumulation. Bottom: Oil Red O staining of neutral lipids (Scale bars, 100 μm). (e) Volcano plot of DEGs of BAT (|log_2_FC| > 2, FDR < 0.05). Highlighted gene: *Fasn*. (f) KEGG pathway enrichment (bubble size: gene count; color: -log_10_[pvalue]). (g) Volcano plot of DEGs of liver (|log_2_FC| > 2, FDR < 0.05). Highlighted gene: *Fasn*. (h) KEGG pathway enrichment of liver (bubble size: gene count; color: -log_10_[pvalue]). (i) Heatmap of lipogenic genes. Relative expression of lipogenic genes in BAT and liver is shown, extending the iWAT analysis presented in Figure 4l. Panels (a-d): Two-tailed unpaired Student’s t-tests.


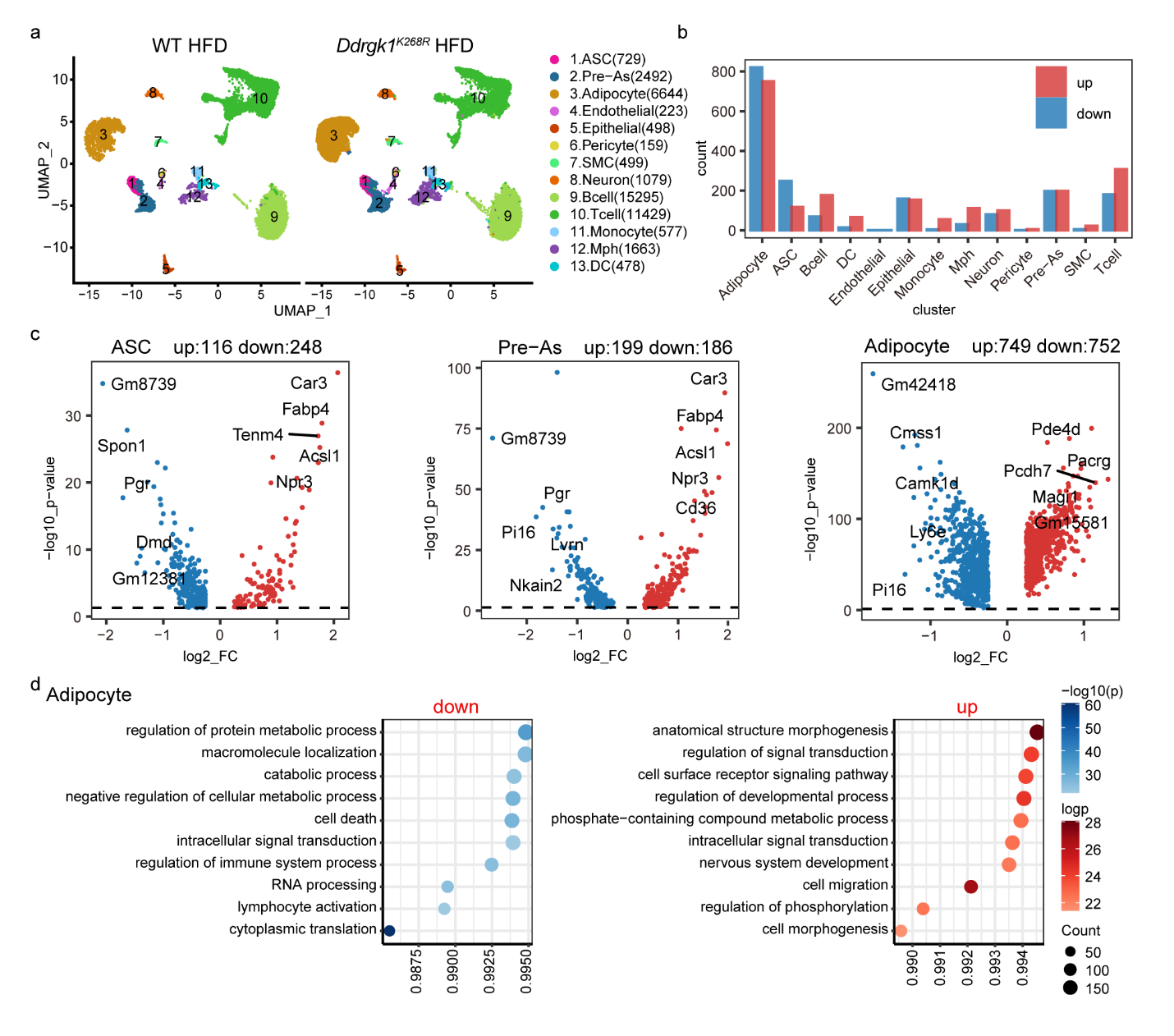


**Extended Data Fig. S7 |** SnRNA-seq analysis of iWAT cellular heterogeneity. (a) Two-dimensional UMAP projection of iWAT, colored by annotated cell types. (b) Cell type-specific differential gene expression between WT and *Ddrgk1^K268R^* mice. Bar plot shows counts of DEGs (|log_2_FC| > 0.5, FDR < 0.05) per cluster. (c) Volcano plots of ASC, Pre-As, Adipocyte DEGs. (d) KEGG pathway enrichment of adipocyte DEGs. Downregulated: Regulation of protein metabolic process. Upregulated: Anatomical structure morphogenesis. Circle size indicates gene count and color shows -log_10_(pvalue).


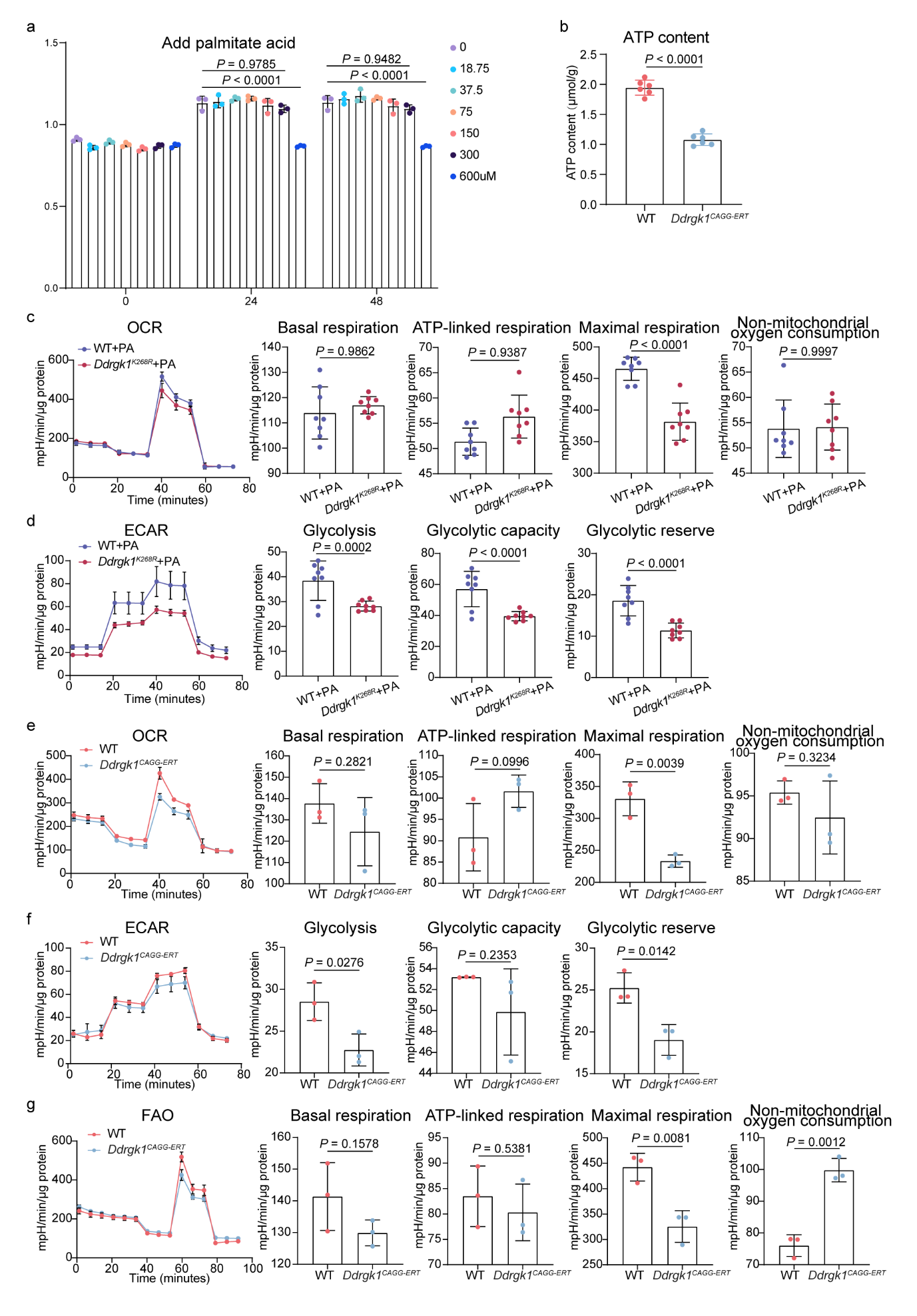


**Extended Data Fig. S8 |** DDRGK1 regulates cellular metabolism. (a) Cell viability of WT SVF cells treated with palmitic acid (PA, 0-600 μM, 0-48 h) assessed by CCK-8 assay. Data are normalized to untreated controls (n = 3 wells). (b) Intracellular ATP levels in *in vitro*-differentiated adipocytes from WT and *Ddrgk1^CAGG-ERT^* mice (n = 6 per group). (c-e) Metabolic flux analysis (Seahorse XFe96) of PA-treated (300 μM, 48 h) adipocytes from WT versus *Ddrgk1^K268R^* mice: (c) OCR under basal, oligomycin, FCCP and rotenone conditions (n = 8 per group). (d) ECAR (n = 8 per group). (e-g) Metabolic flux analysis of differentiated adipocytes from WT versus *Ddrgk1^CAGG-ERT^* mice: (e) OCR under basal, oligomycin, FCCP and rotenone conditions (n = 3 per group). (f) ECAR (n = 8 per group). (g) OCR under FAO conditions with etomoxir, basal, oligomycin, FCCP and rotenone (n = 3 per group). Data in a are shown as mean ± s.d. and analyzed using one-way ANOVA followed by Tukey’s multiple comparison. Data in (b-g) are shown as mean ± s.d. and analyzed using two-tailed unpaired Student’s t-tests.


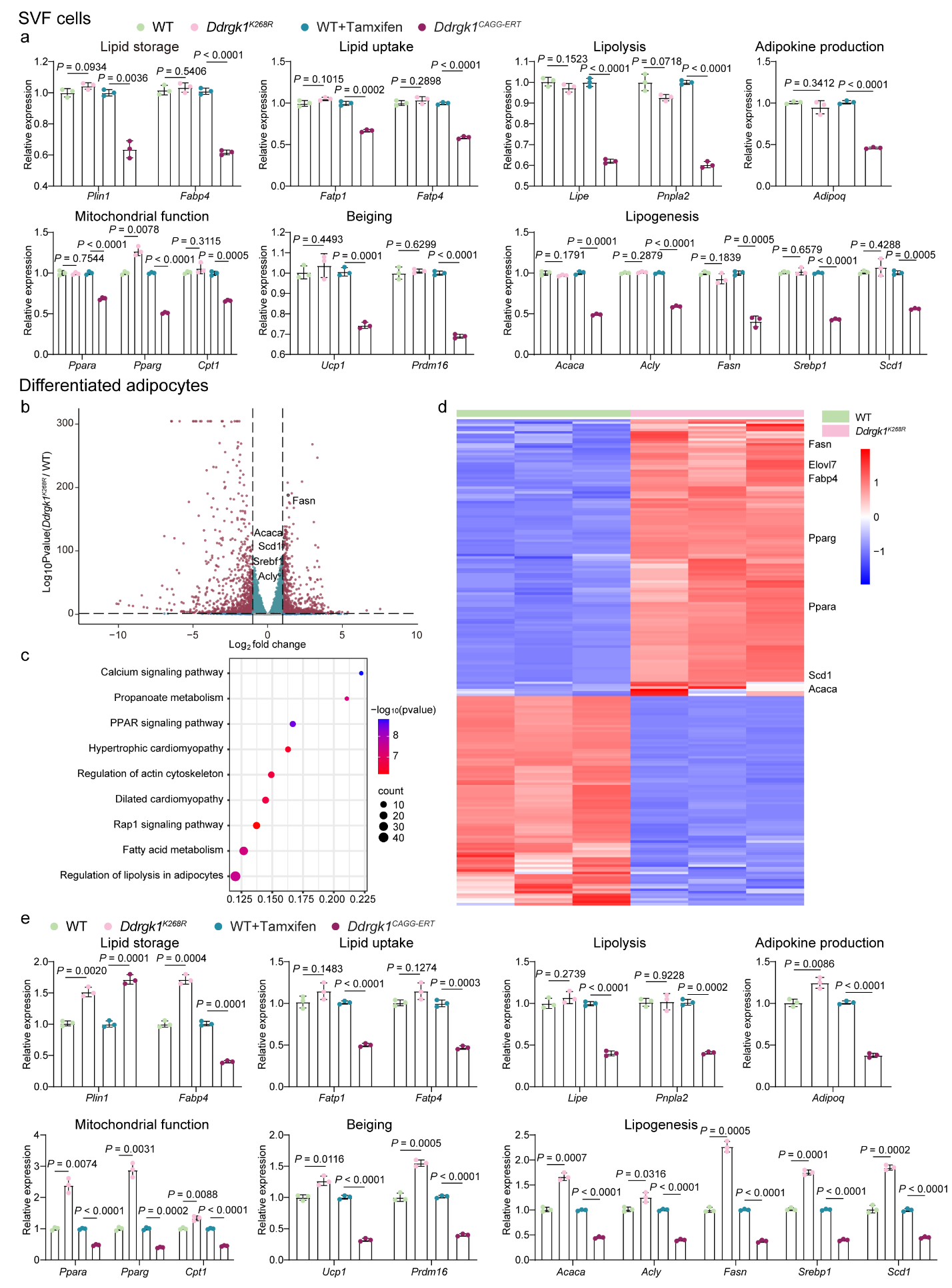


**Extended Data Fig. S9 |** *Ddrgk1^K268R^* impair lipid synthesis, whereas full knockout severely disrupts adipocyte differentiation. (a) Gene expression profiling in SVF cells by RT-qPCR. Functional categories: lipid storage *(Plin1*, *Fabp4*), uptake (*Fatp1*, *Fatp4*), lipolysis (*Lipe*, *Pnpla2*), adipokines (*Adipoq*), mitochondrial function (*Ppara*, *Pparg*, *Cpt1b*), beiging (*Ucp1*, *Prdm16*), *Ddrgk1* and *Fasn*. Data normalized to 36B4 and presented as fold-change vs CD (mean ± SEM, n = 4 biological replicates with 2 technical replicates each). (b-d) Transcriptomic profiling of mature adipocytes. (b) Volcano plot of DEGs (|log_2_FC|>1, FDR<0.05). Highlighted genes: *Acaca, Acly, Fasn, Srebp1c, Scd1*. (c) KEGG pathway enrichment (bubble size: gene count; color: -log_10_[*P*-value]). (d) Heatmap of DEGs. (e) Gene expression profiling in differentiated adipocytes by RT-qPCR. Functional categories: lipid storage *(Plin1*, *Fabp4*), uptake (*Fatp1*, *Fatp4*), lipolysis (*Lipe*, *Pnpla2*), adipokines (*Adipoq*), mitochondrial function (*Ppara*, *Pparg*, *Cpt1b*), beiging (*Ucp1*, *Prdm16*), *Ddrgk1* and *Fasn*. Data normalized to 36B4 and presented as fold-change vs CD (mean ± SEM, n = 4 biological replicates with 2 technical replicates each). In (a-e) data were shown as the mean ± s.d. The statistical significance was calculated using two-tailed unpaired Student’s t-tests.


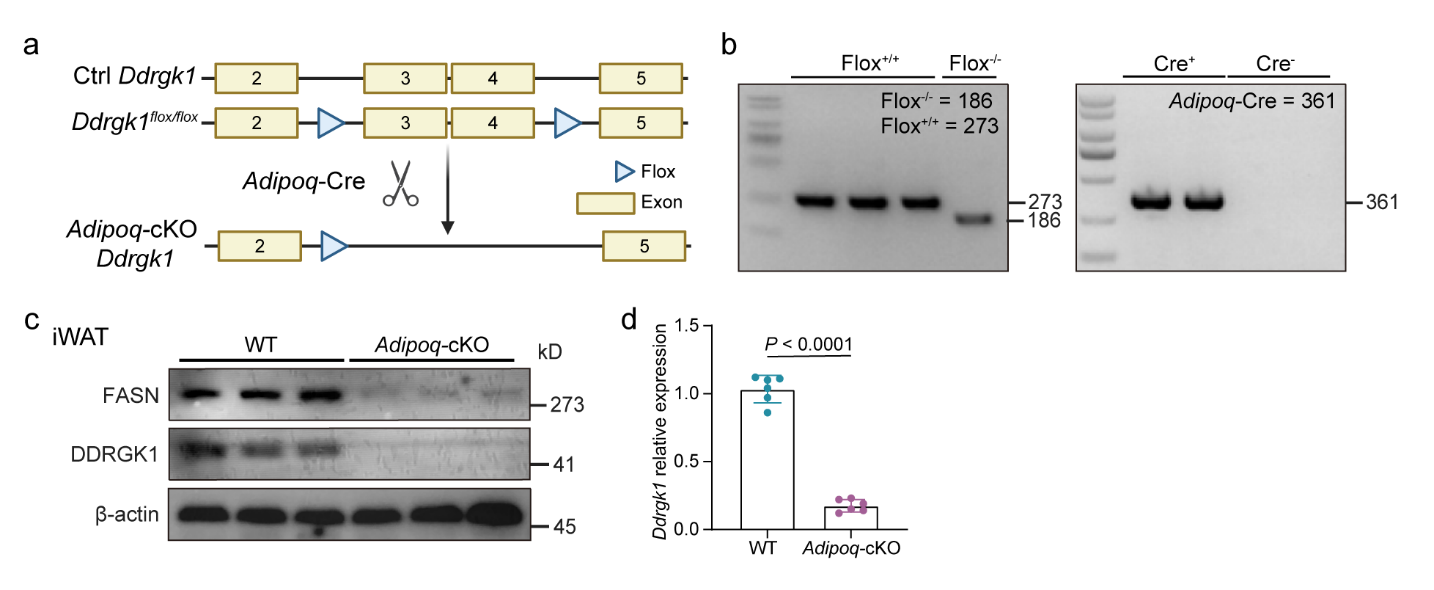


**Extended Data Fig. S10 |** Generation and validation of adipocyte-specific *Ddrgk1* knockout mice. (a) Targeting strategy for adipocyte-specific deletion of DDRGK1. Exons are indicated by numbers, and triangles denote LoxP sites. WT: *Ddrgk1^flox/flox^*; *Adipoq*-cKO: *Ddrgk1^flox/flox^*, *Adipoq*-Cre. (b) Genomic DNA extracted from mouse tail biopsies was subjected to PCR using primers for the *Ddrgk1^flox^* allele and the *Adipoq*-Cre transgene. Agarose gel electrophoresis revealed distinct band patterns corresponding to Flox^-/-^, Flox^+/+^, Cre^+^ and Cre^-^. (c) Western blot analysis of FASN and DDRGK1 protein levels in iWAT (β-actin as loading control). (d) Relative *Ddrgk1* mRNA levels in iWAT detected by RT-qPCR (WT and Adipoq-cKO; n = 6 per group).

Table 1. Patients’ information

| Characteristic | Lean (n=12) | Overweight (n=11) |
| --- | --- | --- |
| Age (years) | 63.83 (9.880) | 65.91 (7.382) |
| Sex |  |  |
| Male | 9 | 6 |
| Female | 3 | 5 |
| Height (m) | 1.692 (0.06952) | 1.633 (0.07837) |
| Weight (kg) | 61.17 (7.779) | 69.15 (7.665) |
| BMI (kg/m2) | 21.31 (1.606) | 25.88 (1.224) |

Table 2. Reagents

| Reagents | Source | Identifier |
| --- | --- | --- |
| ACA ELISA KIT | Coibo | CB11497-Mu |
| MCA ELISA KIT | Coibo | CB11458-Mu |
| Lipofectamine 3000 Reagent | Invitrogen | L3000015 |
| Bodipy 493/503 | Invitrogen | D3922 |
| Palmitic Acid | Kunchuang Biotechnology | KC003 |
| DAPI | MedChemExpress | 28718-91-4 |
| Hoechst 33342 | MedChemExpress | 23491-52-3 |
| Tamoxifen | MedChemExpress | HY-13757A |
| Triglyceride (TG) Content Assay Kit | Sangon | D799796-0100 |
| Pyruvate (PA) Content Assay Kit | Sangon | D799450-0100 |
| Cycloheximide | Selleck | S7418 |
| MG132 | Selleck | S2619 |
| Chloroquine | Selleck | S6999 |
| Anti-DYKDDDDK Tag Immunomagnetic Magnetic Beads | Selleck | B26101 |
| Anti-MYC Tag Immunomagnetic Magnetic Beads | Selleck | B26301 |
| Protein A/G Immunoprecipitation Magnetic beads | Selleck | B23201 |
| Oil Red O | Sigma-Aldrich | O0625 |
| Seahorse XF Cell Mitochondrial Stress Test Kit | Agilent | 103015-100 |
| Seahorse XF Glycolytic Stress Test Kit | Agilent | 103020-100 |
| Seahorse XF Long-Chain Fatty Acid Oxidation Stress Test Kit | Agilent | 103672-100 |
| Fatty Acid Synthase(FAS) Activity Assay Kit | Solarbio | BC0550 |
| The ATP Determination Kit | Invitrogen | A22066 |
| MitoSOX Red | Invitrogen | M36007 |
| MitoTracker Green | Invitrogen | A66441 |

Table 3. Antibodies

| Antibodies | Source | Identifier |
| --- | --- | --- |
| FASN Rabbit mAb (1:1000) | Abcam | ab128870 |
| UFM1 Rabbit mAb (1:1000) | Abcam | ab109305 |
| Ubiquitin Rabbit mAb (1:1000) | Cell Signaling Technology | 43124S |
| Anti-rabbit IgG, HRP-linked Antibody (1:5000) | Cell Signaling Technology | 7074S |
| Anti-mouse IgG, HRP-linked Antibody (1:5000) | Cell Signaling Technology | 7076S |
| Anti-mouse IgG (H+L), F(ab')2 Fragment (Alexa Fluor® 488 Conjugate) (1:5000) | Cell Signaling Technology | 4408S |
| Anti-rabbit IgG (H+L), F(ab')2 Fragment (Alexa Fluor® 488 Conjugate) (1:5000) | Cell Signaling Technology | 4413S |
| PPARγ Rabbit mAb (1:1000) | Cell Signaling Technology | 2435S |
| β-actin Mouse mAb (1:1000) | Cell Signaling Technology | 3700S |
| MYC Rabbit mAb (1:1000) | Cell Signaling Technology | 2276S |
| FALG Rabbit mAb (1:1000) | Cell Signaling Technology | 14793S |
| UCP1 Rabbit mAb (1:200) | Cell Signaling Technology | 72298S |
| F4/80 Rabbit mAb (1:200) | Cell Signaling Technology | 70076S |
| DDRGK1 Rabbit mAb (1:1000) | Proteintech | 21445-1-AP |
| FASN Mouse mAb (1:1000) | Proteintech | 66591-1-Ig |

Table 4. Primer sequences

| Gene m=mouse, h=human | Sequence (5’-3’) |
| --- | --- |
| m*36B4* sen | AGATGCAGCAGATCCGCAT |
| m*36B4* anti | GTTCTTGCCCATCAGCACC |
| m*β-actin* sen | GGCTGTATTCCCCTCCATCG |
| m*β-actin* anti | CCAGTTGGTAACAATGCCATGT |
| m*Fatp1* sen | CGCTTTCTGCGTATCGTCTG |
| m*Fatp1* anti | GATGCACGGGATCGTGTCT |
| m*Fatp4* sen | ACTGTTCTCCAAGCTAGTGCT |
| m*Fatp4* anti | GATGAAGACCCGGATGAAACG |
| m*Glut1* sen | TTCACTGTCGTGTCGCTGTT |
| m*Glut1* anti | TGAGTATGGCACAACCCGC |
| m*Glut4* sen | AGCACCGCAGAGAACACAG |
| m*Glut4* anti | GTCGGGCTTCCAACAGATAG |
| m*Fabp4* sen | TGAAATCACCGCAGACGACA |
| m*Fabp4* anti | CTCTTGTGGAAGTCACGCCT |
| m*Ucp1* sen | GTGAACCCGACAACTTCCGAA |
| m*Ucp1* anti | TGCCAGGCAAGCTGAAACTC |
| m*Prdm16* sen | TCCCACCAGACTTCGAGCTA |
| m*Prdm16* anti | AAAGTCGGCCTCCTTCAGTG |
| m*Cidea* sen | TTCAAGGCCGTGTTAAGGA |
| m*Cidea* anti | CCTTTGGTGCTAGGCTTGG |
| m*Plin1* sen | TGGAGACTGAGGAGAACAAG |
| m*Plin1* anti | ATGTCACAGCCGAGATGG |
| m*Adipoq* sen | TTGTTCCTCTTAATCCTGCCCA |
| m*Adipoq* anti | CCAACCTGCACAAGTTCCCTT |
| m*Atgl* sen | GGATGGCGGCATTTCAGACA |
| m*Atgl* anti | CAAAGGGTTGGGTTGGTTCAG |
| m*Lipe* sen | GCAGGTGGGAATCTCTGCAT |
| m*Lipe* anti | GAGGACTGCAGGGTGGTAAC |
| m*Acaca* sen | GGAGATGTACGCTGACCGAG |
| m*Acaca* anti | TACCCGACGCATGGTTTTCA |
| m*Scd1* sen | TTCTTGCGATACACTCTGGTGC |
| m*Scd1* anti | CGGGATTGAATGTTCTTGTCGT |
| m*Fasn* sen | GCTGCGGAAACTTCAGGAAAT |
| m*Fasn* anti | AGAGACGTGTCACTCCTGGACTT |
| m*Acly* sen | AGTATGGGCTTCATTGGGCACT |
| m*Acly* anti | GGAAATGTCATCCCAGGGGTGA |
| m*Srebpf1c* sen | GGAGCCATGGATTGCACATT |
| m*Srebpf1c* anti | GGCCCGGGAAGTCACTGT |
| m*Ppara* sen | GACAAGGCCTCAGGGTACCA |
| m*Ppara* anti | GCCGAATAGTTCGCCGAAA |
| m*Pparg* sen | TCGCTGATGCACTGCCTATG |
| m*Pparg* anti | GAGAGGTCCACAGAGCTGATT |
| m*Cpt1b* sen | TGAGCCAAACACCACGTTGCCA |
| m*Cpt1b* anti | TAGAGCTCCACGTCATCGGCCA |
| m*Ddrgk1* sen | GAGTACCTGAAACTGAAGGAGG |
| m*Ddrgk1* anti | TGGACTGCTTGATGTAGTTGAT |
| h*β-ACTIN* sen | AGAAGGATTCCTATGTGGGCGAC |
| h*β-ACTIN* anti | AGTACTTGCGCTCAGGAGGA |
| h*FASN* sen | AGGCCTCATAGACCTGCTGA |
| h*FASN* anti | GGGAGATGAGGGGAGTTCCT |

Table 5. IP-MS Analysis

| Protein |
| --- |
| DDX5 |
| KRT1 |
| DDX3X |
| HNRNPH3 |
| HSPA5 |
| KRT2 |
| RPS3A |
| RPS2 |
| FBL |
| NPM1 |
| RPL5 |
| **DDRGK1** |
| HNRNPL |
| RPS13 |
| PKM |
| PTBP1 |
| RPS15A |
| CP |
| LSG1 |
| CHD1 |
| SLC25A5 |
| SDHA |
| CCT5 |
| RPL27 |
| RAN |
| LRRC40 |
| TUBB |
| UQCRC2 |
| DNAJA1 |
| SRSF3 |
| PKP2 |
| CDK1 |
| PABPC1 |
| KRT5 |
| PSMA4 |
| **MAPK1** |
| RPL12 |
| CSK |
| NOP2 |
| **ACLY** |
| PHB2 |
| HNRNPUL1 |
| DDX28 |
| DBR1 |
| SERPINC1 |
| KRT14 |
| SLC3A2 |
| **CDK4** |
| DARS1 |
| PHB1 |
| GRB2 |
| PDIA6 |
| SF1 |
| CHORDC1 |
| TTLL12 |
| HAT1 |
| PSMD3 |
| IDH3B |
| TIPRL |
| HPRT1 |
| CYC1 |
| DNAJB1 |
| RPS15 |
| RPL38 |
| TRAP1 |
| FHL1 |
| PPA1 |
| PHF6 |
| SH3GL1 |
| VDAC3 |
| MRPS18A |
| KRT6A |
| RPS17 |
| KRT16 |
| ARL3 |
| MYL6 |
| YBX1 |
| MRPS21 |
| KRT76 |
| C1QBP |
| TRA2A |
| H3C15 |
| NTPCR |
| SCAMP3 |
| RAB5C |
| GSPT2 |
| IMPDH1 |
| RAB1B |
| EIF3J |
| KRT84 |
| RPS8 |
| NDUFS5 |

Table 6. Metabolic cage data

| Time of Day | Group | Energy Expenditure (kcal/hr) | Pedestrian Locomotion (m/hr) | Distance in Cage Locomotion (m/hr) | Oxygen Consumption (ml/hr) | Carbon Dioxide Production (ml/hr) | Respiratory Exchange Ratio | Locomotor Activity (beam breaks/hr) |
| --- | --- | --- | --- | --- | --- | --- | --- | --- |
| Total | WT | 0.401816161805556 | 124.690839583333 | 174.612604166667 | 82.7940698229167 | 68.2863770104167 | 0.824185460590278 | 119.612847222222 |
| Total | WT | 0.500777953645833 | 115.3664625 | 156.288008333333 | 104.287147708333 | 81.17749075 | 0.778208515451389 | 134.34375 |
| Total | WT | 0.507323014409722 | 183.1807563 | 233.337825 | 105.727531666667 | 81.9627608229167 | 0.775553480902778 | 148.220486111111 |
| Total | WT | 0.432266700868056 | 141.3377375 | 166.886066666667 | 90.37804778 | 68.79459228 | 0.760443037673611 | 89.1631944444444 |
| Total | WT | 0.420895871701389 | 114.9677713 | 159.6310231 | 88.4179427083333 | 65.4979734583333 | 0.740058052777778 | 118.756944444444 |
| Total | WT | 0.463701526215278 | 98.01396875 | 152.976285416667 | 95.82624156 | 77.8031718229167 | 0.811212561458333 | 182.203125 |
| Total | *Ddrgk1^K268R^* | 0.526172579513889 | 154.320520833333 | 207.164520833333 | 109.5691022 | 85.3171290729167 | 0.779212583159722 | 176.463541666667 |
| Total | *Ddrgk1^K268R^* | 0.478878516493056 | 114.7726 | 155.3939625 | 100.136118229167 | 76.16824163 | 0.760893238541667 | 140.458333333333 |
| Total | *Ddrgk1^K268R^* | 0.428265684 | 66.62195625 | 103.695222916667 | 88.9312468229167 | 70.3324025833333 | 0.790333370486111 | 126.793402777778 |
| Total | *Ddrgk1^K268R^* | 0.470696380381944 | 78.1999625 | 127.7726188 | 97.6681080208333 | 77.5645273333333 | 0.793869237152778 | 147.756944444444 |
| Total | *Ddrgk1^K268R^* | 0.4842604015625 | 120.778279166667 | 156.687808333333 | 100.440035833333 | 79.9513726979167 | 0.794886359895833 | 119.414930555556 |
| Total | *Ddrgk1^K268R^* | 0.546516182465278 | 123.672745833333 | 182.7363375 | 113.5450016 | 89.54370078 | 0.787225600347222 | 164.227430555556 |
| Dark | WT | 0.421743787847222 | 114.281720833333 | 159.787108333333 | 86.89807513 | 71.6803554166667 | 0.824845776736111 | 135.152777777778 |
| Dark | WT | 0.532109002777778 | 118.061633333333 | 157.531108333333 | 111.118541041667 | 85.16350138 | 0.765390575 | 169.836805555556 |
| Dark | WT | 0.457826225694444 | 176.372929166667 | 223.685895833333 | 115.998953333333 | 89.0350863541667 | 0.767384348611111 | 225.434027777778 |
| Dark | WT | 0.473020689930556 | 138.905608333333 | 163.712 | 99.0341958333333 | 74.7983085208333 | 0.754401815972222 | 139.298611111111 |
| Dark | WT | 0.464556707638889 | 105.536183333333 | 145.988682916667 | 97.58724813 | 72.3014187083333 | 0.740151514236111 | 184.6875 |
| Dark | WT | 0.479654163194444 | 93.0045791666666 | 143.8642875 | 99.3908235416667 | 79.5252475416667 | 0.798684493402778 | 211.513888888889 |
| Dark | *Ddrgk1^K268R^* | 0.573508150694444 | 151.0019625 | 202.385408333333 | 119.915052708333 | 91.2503811458333 | 0.760486078819444 | 255.072916666667 |
| Dark | *Ddrgk1^K268R^* | 0.515550722569444 | 116.2990875 | 157.0104625 | 108.378455 | 79.9559017916667 | 0.736721778472222 | 189.059027777778 |
| Dark | *Ddrgk1^K268R^* | 0.555624683680556 | 63.6672833333333 | 98.835475 | 95.0180636458333 | 75.3707350208333 | 0.792963078 | 162.934027777778 |
| Dark | *Ddrgk1^K268R^* | 0.501158984 | 74.5668 | 121.585070833333 | 104.088607291667 | 82.2294573958333 | 0.789975253 | 188.28125 |
| Dark | *Ddrgk1^K268R^* | 0.493265514583333 | 110.121958333333 | 142.735420833333 | 102.246621041667 | 81.6560391666667 | 0.797802034027778 | 136.28125 |
| Dark | *Ddrgk1^K268R^* | 0.544006702777778 | 115.445154166667 | 170.725845833333 | 113.080064166667 | 88.9314390416667 | 0.785907016 | 168.572916666667 |
| Light | WT | 0.381888535763889 | 135.099958333333 | 189.4381 | 78.6900645208333 | 64.8923986041667 | 0.823525144444444 | 104.072916666667 |
| Light | WT | 0.469446904513889 | 112.671291666667 | 155.044908333333 | 97.45575438 | 77.19148013 | 0.791026455902778 | 98.8506944444444 |
| Light | WT | 0.459021345138889 | 189.988583333333 | 242.989754166667 | 95.45611 | 74.8904352916667 | 0.783722613194444 | 71.0069444444444 |
| Light | WT | 0.391512711805556 | 143.769866666667 | 170.060133333333 | 81.7218997291667 | 62.7908760416667 | 0.766484259 | 39.0277777777778 |
| Light | WT | 0.377235035763889 | 124.399359166667 | 173.273363333333 | 79.2486372916667 | 58.6945282083333 | 0.739964591319444 | 52.8263888888889 |
| Light | WT | 0.447748889236111 | 103.023358333333 | 162.088283333333 | 92.2616595833333 | 76.0810961041667 | 0.823740629513889 | 152.892361111111 |
| Light | *Ddrgk1^K268R^* | 0.478837008333333 | 157.639079166667 | 211.943633333333 | 99.2231516666667 | 79.383877 | 0.797939088 | 97.8541666666667 |
| Light | *Ddrgk1^K268R^* | 0.442206310416667 | 113.2461125 | 153.7774625 | 91.8937814583333 | 72.3805814583333 | 0.785064698611111 | 91.8576388888889 |
| Light | *Ddrgk1^K268R^* | 0.398705143055556 | 69.5766291666667 | 108.554970833333 | 82.84443 | 65.2940701458333 | 0.787703662847222 | 90.6527777777778 |
| Light | *Ddrgk1^K268R^* | 0.440233776388889 | 81.833125 | 133.960166666667 | 91.24760875 | 72.8995972708333 | 0.797763221180556 | 107.232638888889 |
| Light | *Ddrgk1^K268R^* | 0.475255288541667 | 131.4346 | 170.640195833333 | 98.63345063 | 78.2467062291667 | 0.791970685763889 | 102.548611111111 |
| Light | *Ddrgk1^K268R^* | 0.549025662152778 | 131.9003375 | 194.746829166667 | 114.009938958333 | 90.1559625208333 | 0.788544185069444 | 159.881944444444 |

Table 7. ANCOVA results

| Effect | Full day | | Light | | Dark | |
| --- | --- | --- | --- | --- | --- | --- |
|  | Fat mass | Group | Fat mass | Group | Fat mass | Group |
| Food Consumed (kcal/period) | 0.0321* | 0.1292 | 0.0766 | 0.0415* | 0.0437* | 0.3434 |
| Water Consumed (ml/period) | 0.3694 | 0.2185 | 0.7060 | 0.3633 | 0.2661 | 0.2213 |
| Energy Expenditure(kcal/period) | 0.0144* | 0.0248* | 0.0055** | 0.0088* | 0.0772 | 0.1238 |
| Oxygen Consumption(ml/hr) | 0.0150 * | 0.0266* | 0.0053** | 0.0085* | 0.0822 | 0.1368 |
| Carbon Dioxide Production (ml/hr) | 0.0178* | 0.0255* | 0.0084** | 0.0130* | 0.0717 | 0.0904 |

Data are presented as mean ± SEM. * *P* < 0.05, ** *P* < 0.01, *** *P* < 0.001.

Table 8. Proteomic profiling of down-regulated and UFMylation-associated proteins in iWAT

| Protein | WT | *Ddrgk1^K268R^* | Fold change | *P*-value |
| --- | --- | --- | --- | --- |
| IGKV8-28 | 4.585311086 | 9.125468178 | 1.990152469 | 0.044746355 |
| IGHV1-43 | 0.323815384 | 0.729653604 | 2.253301236 | 0.032171695 |
| IGKV4-53 | 0.614955721 | 1.183267682 | 1.924151027 | 0.011869139 |
| IGHA | 2.880957287 | 6.458576803 | 2.24181623 | 0.048131853 |
| IGKV16-104 | 0.604293222 | 1.032362083 | 1.708379384 | 0.010649424 |
| IGKV5-43 | 0.081785356 | 0.267933082 | 2.18403469 | 0.046554674 |
| IGHV9-3 | 0.539254029 | 0.885410998 | 1.641918187 | 0.047663512 |
| METTL27 | 0.450897457 | 0.697296758 | 1.546464163 | 0.040560598 |
| IGKV12-46 | 1.002959112 | 1.810715389 | 1.805373088 | 0.024963655 |
| KIDINS220 | 0.146114043 | 0.173426631 | 1.780389757 | 0.031234151 |
| OBP1B | 0.267337217 | 0.648805553 | 2.426918185 | 0.023238809 |
| CNTRL | 0.238672365 | 0.440413407 | 1.845263509 | 0.021871469 |
| SCGB1B29 | 0.491906971 | 1.070148452 | 2.175509834 | 0.006238688 |
| BIN2 | 0.983882931 | 2.646339639 | 2.689689551 | 0.042410964 |
| ARHGAP45 | 0.256764292 | 0.689543254 | 2.685510705 | 0.025262696 |
| C130026I21RIK | 0.259297856 | 0.544364145 | 2.099377733 | 0.042077584 |
| SLC4A1AP | 0.382414063 | 0.579984465 | 1.516640003 | 0.039214999 |
| MORC3 | 0.248294841 | 0.426729999 | 1.718642229 | 0.037361999 |
| HEATR1 | 0.277544099 | 0.544093523 | 1.960385842 | 0.028915054 |
| CNN1 | 2.067667708 | 0.743240826 | 0.359458545 | 0.014558206 |
| ECI3 | 0.278181818 | 0.126037516 | 0.453076038 | 0.021254445 |
| SEMA4D | 0.091544468 | 0.342448071 | 3.740783899 | 0.022260202 |
| RPL21 | 1.062864418 | 1.828355478 | 1.720215154 | 0.026794241 |
| IFI203 | 0.317655228 | 0.80472847 | 2.533339288 | 0.009789216 |
| SFN | 0.412682332 | 0.989497242 | 2.397721357 | 0.046038544 |
| SP3 | 0.098016988 | 0.195701592 | 1.996608909 | 0.029399609 |
| PARG | 0.092725044 | 0.144398498 | 1.55727614 | 0.034933373 |
| RPP30 | 0.285443877 | 0.548119604 | 1.920235984 | 0.016829991 |
| CORO1A | 4.668062263 | 13.31188024 | 2.851692949 | 0.035490734 |
| IGKV12-41 | 2.271694333 | 4.481839354 | 1.972905988 | 0.036130405 |
| KLK1B16 | 0.20704462 | 1.323719788 | 6.39340345 | 0.011060929 |
| PTPRC | 5.81155389 | 14.80008988 | 2.546666547 | 0.038307604 |
| LTF | 0.868072495 | 4.421890925 | 5.093918942 | 0.03513062 |
| HMGN2 | 3.965881194 | 8.957324809 | 2.258596355 | 0.001010273 |
| SUB1 | 3.758838464 | 7.037803781 | 1.872334725 | 0.045569971 |
| ACACB | 2.896263859 | 1.35164255 | 0.46668488 | 0.030857088 |
| CD79A | 0.399125479 | 1.115455323 | 2.794748474 | 0.042879782 |
| GUSB | 0.725720424 | 1.288370077 | 1.775298081 | 0.029808637 |
| SLC1A3 | 1.260150189 | 0.613242627 | 0.486642491 | 0.042002052 |
| H2-AA | 1.063627771 | 3.657367302 | 3.438578233 | 0.004732102 |
| H2-AB1 | 1.151017901 | 2.592758368 | 2.252578666 | 0.00872557 |
| CD44 | 3.758779874 | 7.469572662 | 1.987233334 | 0.031442822 |
| ARL4A | 0.480051046 | 0.241896249 | 0.50389693 | 0.031155098 |
| LSP1 | 5.000771843 | 15.85504478 | 3.170519526 | 0.024666889 |
| MCM3 | 0.385375673 | 1.114660214 | 2.892399007 | 0.02633192 |
| H2-DMA | 0.527108005 | 1.298647593 | 2.463722009 | 0.045989864 |
| ECHDC2 | 1.761147611 | 0.888255041 | 0.504361495 | 0.011435038 |
| PTPN6 | 3.664410477 | 8.608197821 | 2.349135795 | 0.031846598 |
| HMGB2 | 3.314153442 | 11.00817616 | 3.321565026 | 0.023039244 |
| CEACAM1 | 0.14211192 | 0.254950321 | 1.794010817 | 0.047667338 |
| **FASN** | 243.1996785 | 138.1493659 | 0.568049131 | 0.041966452 |
| **DDRGK1** | 0.274739866 | 0.339960226 | 1.237389501 | 0.072842559 |
| **UFL1** | 1.369618252 | 1.442782723 | 1.053419609 | 0.594869651 |
| **UFSP2** | 0.434957218 | 0.372615889 | 1.285008756 | 0.43696923 |
| **UFC1** | 1.120936788 | 1.221912084 | 1.090081169 | 0.567027594 |
| **UFM1** | 4.309252984 | 5.477105016 | 1.268606172 | 0.033899092 |
| **CYB5R3** | 7.000937167 | 6.477943983 | 0.925296689 | 0.566719016 |
| **CDK5RAP3** | 0.620405023 | 0.683643178 | 1.101930438 | 0.276090334 |
